# Temporal change in prevalence of BMI categories in India: patterns across States and Union territories of India, 1999–2021

**DOI:** 10.1186/s12889-024-18784-4

**Published:** 2024-05-16

**Authors:** Meekang Sung, Akhil Kumar, Raman Mishra, Bharati Kulkarni, Rockli Kim, S. V. Subramanian

**Affiliations:** 1 Department of Social and Behavioral Sciences, Harvard T. H. Chan School of Public Health, Boston, MA USA; 2 Faculty of Arts and Sciences, University of Toronto, Toronto, ON Canada; 3 Interdisciplinary Program in Precision Public Health, Department of Public Health Sciences, Graduate School of Korea University, Seoul, Republic of Korea; 4 Division of Reproductive & Child Health & Nutrition, Indian Council of Medical Research, V. Ramalingaswami Bhawan, Ansari Nagar, New Delhi, India; 5 Division of Health Policy and Management, College of Health Science, Korea University, Seoul, Republic of Korea; 6 Harvard Center for Population and Development Studies, 9 Bow Street, Cambridge, MA 02138 USA

**Keywords:** States/Union territories, India, Body mass index, Double burden of malnutrition, Malnutrition, Obesity, Change

## Abstract

**Background:**

The problem of overweight/obesity often coexists with the burden of undernutrition in most low- and middle-income countries. BMI change in India incorporating the most recent trends has been under-researched.

**Methods:**

This repeated cross-sectional study of 1,477,885 adults in India analyzed the prevalence of different categories of BMI among adults (age 20–54) in 4 rounds of National Family Health Surveys (1998–1999, 2005–2006, 2015–2016, and 2019–2021) for 36 states/UTs. State differences across time were harmonized for accurate analysis. The categories were Severely/Moderately Thin (BMI < 17.0), Mildly Thin (17.0-18.4), Normal (18.5–24.9), Overweight (25.0-29.9), and Obese (≥ 30.0). We also estimated change in Standardized Absolute Change (SAC), ranking of states, and headcount burden to quantify the trend of BMI distribution across time periods for all-India, urban/rural residence, and by states/UTs.

**Results:**

The prevalence of thinness declined from 31.7% in 1999 to 14.2% in 2021 for women, and from 23.4% in 2006 to 10.0% in 2021 for men. Obesity prevalence increased from 2.9% (1999) to 6.3% (2021) for women, and from 2.0% (2006) to 4.2% (2021) for men. In 2021, the states with the highest obesity prevalence were Puducherry, Chandigarh, and Delhi. These states also had a high prevalence of overweight. Dadra and Nagar Haveli and Diu, Gujarat, Jharkhand, and Bihar had the highest prevalence of severe/moderately thin. Prevalence of extreme categories (severely/moderately thin and obese) was larger in the case of women than men. While States/UTs with a higher prevalence of thin populations tend to have a larger absolute burden of severe or moderate thinness, the relationship between headcount burden and prevalence for overweight and obese is unclear.

**Conclusions:**

We found persistent interstate inequalities of undernutrition. Tailored efforts at state levels are required to further strengthen existing policies and develop new interventions to target both forms of malnutrition.

**Supplementary Information:**

The online version contains supplementary material available at 10.1186/s12889-024-18784-4.

## Background

 The Sustainable Development Goal (SDG) 2 seeks to end hunger and ensure access to safe, nutritious, and sufficient food year-round by 2030. The SDG 3 aims to ensure healthy lives and promote wellbeing for all at all ages [1]. It is important to evaluate the nutritional status to devise effective policies to ascertain these goals. Body Mass Index (BMI) serves as a good metric for evaluating population-level nutritional status and future health risks. Also, the widespread and longstanding application of BMI contributes to its utility at the population level [2]. 

It is increasingly being recognized that the emerging problem of overweight often coexists with the burden of undernutrition in most low- and middle-income countries (LMIC), causing a double burden of malnutrition [3]. This is mostly because of the persistent rise in overweight and obesity over the past few decades [4]. More people are exposed to unhealthy diets, which include readily available, less nutritious, and highly processed foods and beverages, as well as reduced physical activity, all of which increase the risk of overweight and obesity in LMIC [5]. The growing evidence of the Double Burden of Malnutrition (DBM) suggests that comprehensive policy efforts are needed to address the issues of undernutrition and obesity simultaneously.

Various biological factors (such as age and sex), socioeconomic status (individual and neighborhood wealth), and several demographic and environmental factors (urban residence, food environment, and local-level economic development) consistently affect the distribution of malnutrition [6–10]. Some previous studies have also documented that, in most developing countries, malnutrition tends to be clustered in specific geographical regions [8, 11, 12]. 

The significant impact of state roles on health outcomes in India underscores the necessity of conducting state-level analyses. As a federation comprising 28 states and 8 Union Territories (UTs) [13], India delegates the responsibility for developing social sector policies, including nutrition, to the individual state and UT governments [14, 15]. These entities are pivotal in governance, administration, and delivering social welfare services like healthcare, education, welfare schemes, and infrastructure development. They possess the autonomy to craft and execute initiatives suited to their unique local contexts. Consequently, the distinctiveness of the policy process at the state level in India is a key factor in explaining the variations across states.

Previous studies on the distribution of BMI trends in India have reported emerging DBM [16–20] with severe thinness decreasing and obesity increasing [17, 21, 22]. However, the studies did not review the most recent data of 2019–2021 of the National Family Health Survey (NFHS-5) or did not analyze the change in distribution over 20 years using a representative public data set. There is also a dearth of literature focusing on the state-level difference in BMI outcomes. (Additional file 1, Appendix S1).

In this study, we present an up-to-date and comprehensive description of the trends in the prevalence of different BMI categories among adults in India and its 36 states/UTs between 1999 and 2021. We used data from a sizable, nationally representative sample from repeated rounds of NFHS. We also estimated the absolute headcount burden of BMI outcomes for India and each of the states/UTs for its importance from a policy perspective [23]. 

## Methods

### Data

The study used repeated cross-sectional data from four waves of the NFHS covering all states and UTs in India [24–27]. The four surveys were conducted in 1998-99, 2005-06, 2015-16 and 2019-21, hereafter identified with the end year of each survey. All rounds employed a multistage stratified cluster sampling design and used the latest available Census of India at the time of the survey as their sampling frame. The computer-assisted personal interviewing methods and frequent use of field check tables helped NFHS to run extensive data quality checks and go through real-time feedback, minimizing errors in data recording. The sampling methods are discussed in detail in Additional file 1, Appendix S2. Men’s data was collected only in the subsample of households selected for the state model resulting in substantial differences in sample size [27]. 

Weight and height for BMI calculation were measured through the biomarker questionnaire since 2006. In 1999, only women’s height and weight were recorded without a separate biomarker questionnaire. The Seca 874 digital scale was used to measure weight, and the Seca 213 stadiometer was used for measuring height [27]. The responses from the biomarker questionnaire, including BMI data, are included in both the Individual Recode dataset (1999–2021) and Household Member Recode dataset (2006–2021) for women, while information on childbirth (relevant for our study for defining the study population) is captured only in the Individual Recode dataset. To ensure consistent data analysis, we, therefore, utilized the individual recode dataset for women. Even though the two datafiles that provide the BMI data have small differences in terms of the sample size, the percentage prevalence of the sample falling in each of the BMI category (the outcome of interest in our study) was near-identical (Table S1). We utilized the Household Member Recode dataset for men as BMI data was not present in the individual interview data for men.

### Study population

The study population was adult women aged 20–49 years who were not currently pregnant and had not given birth in the last two months, and men aged 20–54 years who lived in households that were selected for the state module. Men’s data was not collected in the 1998-99 round, limiting the study population to only women in 1998-99. The upper limit of age is determined by the NFHS survey design. Observations for which BMI measurements were reported as “don’t know” or were missing (unreported) were excluded.

### Outcome

Based on the BMI cutoffs of WHO [28] and definitions of chronic energy deficiency of the International Dietary Energy Consultative Group [29], the BMI outcomes were divided into five categories: Severe/Moderate Thinness (BMI < 17.0), Mild Thinness (17.0 -18.4), Normal (18.5 - 24.9), Overweight (25.0- 29.9), and Obese (≥30.0). These categories are also used for Demographic Health Survey reports [30]. 

### Constructing comparable state estimates

While currently there are 28 states and 8 UTs, there have been changes in these numbers due to geometric changes of some of the states and redefinition of UTs and states. In 1998 there were 26 states and 7 UTs. Chhattisgarh, Uttarakhand, and Jharkhand were formed in 2000 and Telangana was created in 2016. Due to these changes, it is difficult to create a cross-sectional panel of states and UTs that is repeated over time. We used the methodology used in recent publications to solve this problem [31, 32], assigning surveyed districts in older survey years to their current states (Additional file 1, Appendix S3).

### Analysis

We calculated the percentage of BMI outcomes at each of the four time periods to estimate trends over time for all-India, place of residence (urban/rural), and states/UTs. The prevalence estimates used the individual weights from the survey to account for the multi-stage stratified cluster sampling design. We calculated the Standardized Absolute Change (SAC) to quantify the change in BMI outcomes across time periods in percentage points. For example, SAC for each district during the period between 2016 and 2021 was computed as $\text{S}\text{A}\text{C}=\frac{{P}_{t}-{P}_{x}}{2021-2016}=\frac{{P}_{t}-{P}_{x}}{5}$; where, ${P}_{t}$ refers to the prevalence in recent year (e.g., 2021), ${P}_{x}$represents the prevalence in a previous year in consideration (e.g., 2016). A negative SAC value indicates a declining prevalence over time whereas a positive SAC value indicates rising prevalence during this period.

We used box plots and heat tables to assess the extent to which state inequalities in BMI outcomes have increased/decreased over time. Descriptive assessments of the state-level patterns over time were made using scatterplots and correlations. Specifically, we examined whether the magnitude and patterns of change are correlated with the prevalence of BMI outcomes in 1999 (women) or 2006 (men), which were each considered as a baseline.

We estimated the current population headcounts of BMI categories for India and for the states/UTs in 2021 using the microdata and Census of India Population Projections [33]. We used the methodology provided by Integrated Public Use Microdata Series (IPUMS) [34] with appropriate modifications for our purposes (Additional file 1, Appendix S4). To account for proportion of women who were currently pregnant or recently gave birth within the last 2 months, we assumed that the proportion in NFHS-5 is similar to the 2021 population projections.

The software STATA 15.0 [35] and Excel [36] were used for computations and visualization.

## Results

### Sample characteristics

The study population was 1,281,498 non-pregnant women aged 20–49 years and 270,814 men aged 20–54 years. The percentage of missing or implausible BMI was ranged from 1.7 to 7.1% for women and from 13.7 to 26.5% for men. The final analytic sample after exclusion for women was 71,385 (1999), 90,333 (2006), 531,433 (2016), and 551,027 (2021). For men it was 58,935 (2006), 92,574 (2016), and 82,198 (2021) (Table 1).

**Table 1:** Study sample size selection from the four National Family Health Surveys (NFHS), 1999–2021

Survey round (Year)	Sample size based on inclusion criteria (*n*)		Missing or implausible values (*n* , (%))		Final study sample size	
	Women	Men	Women	Men	Women	Men
NFHS-2(1998-99)	76,880	-	5,495 (7.1)	-	71,385	-
NFHS-3(2005-06)	94,575	74,572	4,242 (4.5)	15,637 (26.5)	90,333	58,935
NFHS-4(2015-16)	540,840	105,351	9,407 (1.7)	12,777 (13.8)	531,433	92,574
NFHS-5(2019-21)	569,203	95,726	18,176 (3.2)	13,528 (16.5)	551,027	82,198
**All waves**	**1,281,498**	**270,814**	**37,320 (2.9)**	**37,107 (13.7)**	**1,244,178**	**233,707**

### Patterns of change in BMI outcomes

**Fig. 1:**
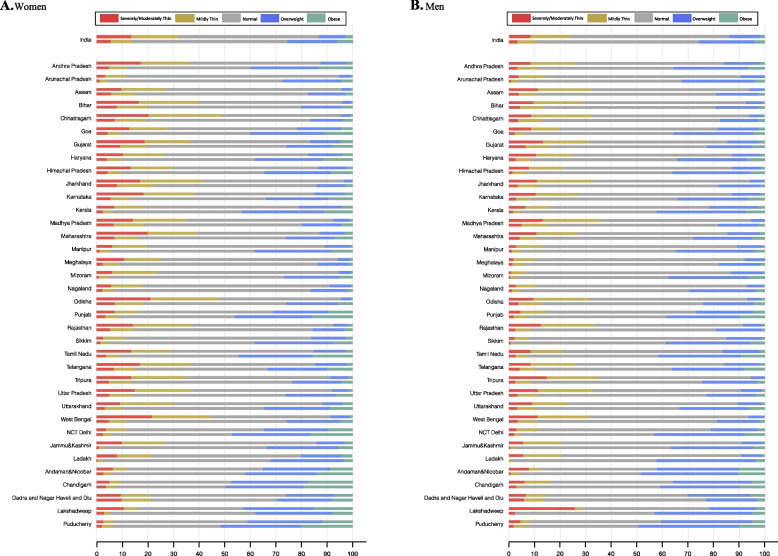
Comparative Distribution of Body Mass Index (BMI) Across States and Union Territories: Earliest and Latest Survey Period (2021). In both panels (**A**) and (**B**), the upper bar represents the BMI distribution for earliest period, while the lower bar depicts the BMI distribution for the latest period (2021). The cutoff points of BMI categories are: Severely/Moderately Thin (<17.0), Mildly Thin (16.0-18.4), Normal (18.5-24.9), Overweight (25.0-29.9), and Obese ( ≥30.0)** A** Women. The earliest survey period for Ladakh is 2006. The earliest survey period for Andaman & Nicobar, Chandigarh, Dadra and Nagar Haveli and Diu, Lakshadweep, and Puducherry is 2016. The earliest survey period for all other states is 1999.** B **Men. The earliest survey period for Andaman & Nicobar, Chandigarh, Dadra and Nagar Haveli and Diu, Lakshadweep, and Puducherry is 2016. The earliest survey period for all other states is 2006

**Fig. 2:**
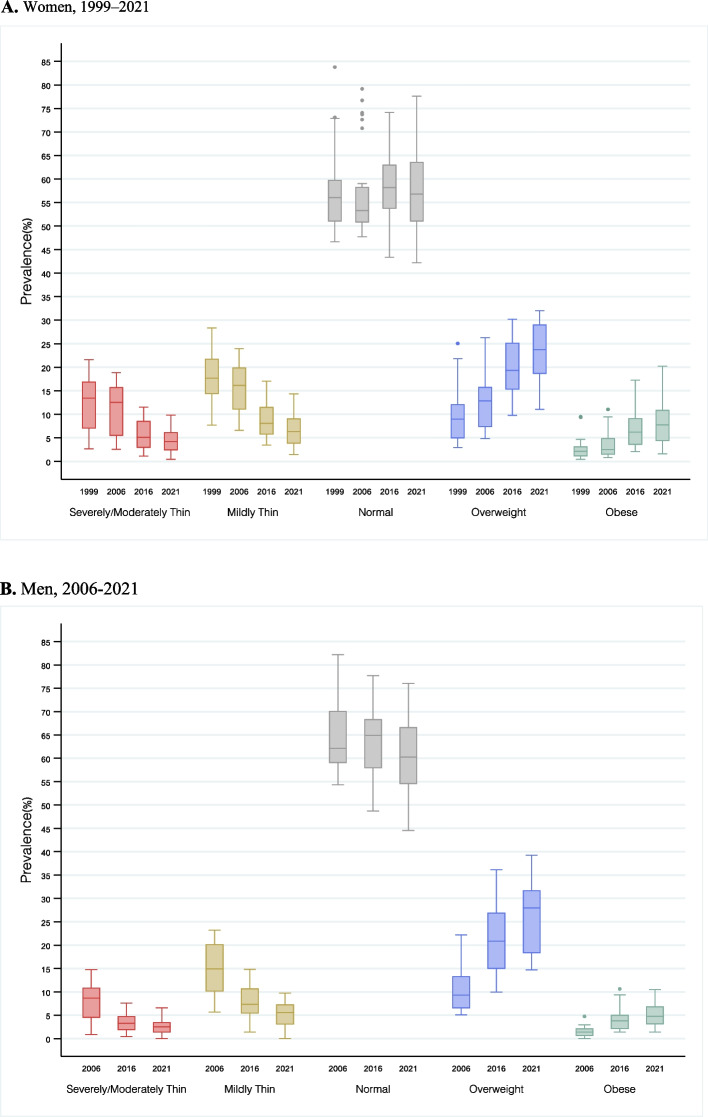
Summary distribution of state/Union Territory-level Body Mass Index (BMI) category. For both panels (**A**) and (**B**), the box-and-whisker plots shows the variability of a data set using lowest and highest values, and quartiles of the data. The upper and lower whiskers represent minimum and maximum values, respectively. The upper outline of the box depicts the 75th and the lower outline the 25th percentile, respectively. The line within the box (separating the darker and lighter tones of grey) shows the median (i.e., 50th percentile).** A **Women, 1999–2021. **B** Men, 2006-2021

The prevalence of severely/moderately thin or mildly thin (hereafter referred to as thinness when the two outcomes are added) in men and women has steadily decreased while the prevalence of overweight/obesity has increased over the years. Thinness prevalence declined from 31.7% in 1999 to 14.2% in 2021 in the case of women, and from 23.4% in 2006 to 10.0% in 2021 in the case of men. Overweight/obesity increased from 13.1% in 1999 to 25.7% in 2021 for women, and from 13.9% in 2006 to 25.9% in 2021 for men (Table 2; Figs. 1 and 2). Overall, thinness was more prevalent for women across all survey rounds.

**Table 2:** Sample size (n) and prevalence (%) of Body Mass Index (BMI) categories by year

	Women				Men		
**BMI Category (Weight divided by Height^2)**	**1999 (***n*** = 71,385)**	**2006 (***n*** = 90,333)**	**2016 (***n*** = 531,433)**	**2021 (***n*** = 551,027)**	**2006 (***n*** = 58,935)**	**2016 (***n*** = 92,574)**	**2021 (***n*** = 82,198)**
**Severely /Moderately Thin (< 17.0)**							
All	9,647 (13.5%)	10,384 (11.5%)	38,536 (7.3%)	30,424 (5.5%)	4,909 (8.3%)	4,296 (4.6%)	2,645 (3.2%)
Urban	1,958 (8.4%)	3,296 (8.0%)	7,165 (4.5%)	4,906 (3.5%)	1,967 (6.7%)	1,070 (3.7%)	517 (2.5%)
Rural	7,689 (16.0%)	7,088 (14.5%)	31,371 (8.4%)	25,518 (6.2%)	2,942 (10.0%)	3,226 (5.1%)	2,128 (3.5%)
**Mildly Thin (17.0-18.4)**							
All	12,963 (18.2%)	13,683 (15.1%)	60,255 (11.3%)	47,791 (8.7%)	8,925 (15.1%)	9,094 (9.8%)	5,611 (6.8%)
Urban	2,736 (11.7%)	4,327 (10.5%)	11,300 (7.1%)	7,554 (5.4%)	3,397 (11.5%)	1,934 (6.7%)	955 (4.5%)
Rural	10,227 (21.3%)	9,356 (19.1%)	48,955 (13.1%)	40,237 (9.8%)	5,528 (18.7%)	7,160 (11.3%)	4,656 (7.6%)
**Normal (18.5–24.9)**							
All	39,401 (55.2%)	49,755 (55.1%)	316,746 (59.6%)	331,220 (60.1%)	36,914 (62.6%)	60,348 (65.2%)	52,690 (64.1%)
Urban	12,721 (54.4%)	22,273 (53.9%)	87,600 (55.2%)	75,737 (54.6%)	18,159 (61.7%)	17,547 (60.4%)	12,228 (58.0%)
Rural	26,680 (55.6%)	27,482 (56.1%)	229,146 (61.5%)	255,483 (62.0%)	18,755 (63.6%)	42,801 (67.4%)	40,462 (66.2%)
**Overweight (25.0-29.9)**							
All	7,304 (10.2%)	12,624 (14.0%)	88,673 (16.7%)	106,652 (19.4%)	7,027 (11.9%)	16,016 (17.3%)	17,839 (21.7%)
Urban	4,462 (19.1%)	8,433 (20.4%)	37,978 (23.9%)	35,294 (25.4%)	4,998 (17.0%)	7,057 (24.3%)	6,004 (28.5%)
Rural	2,842 (5.9%)	4,191 (8.6%)	50,695 (13.6%)	71,358 (17.3%)	2,029 (6.9%)	8,959 (14.1%)	11,835 (19.4%)
**Obese (≥ 30.0)**							
All	2,070 (2.9%)	3,887 (4.3%)	27,223 (5.1%)	34,940 (6.3%)	1,160 (2.0%)	2,820 (3.0%)	3,413 (4.2%)
Urban	1,502 (6.4%)	3,000 (7.3%)	14,693 (9.3%)	15,222 (11.0%)	914 (3.1%)	1,429 (4.9%)	1,382 (6.6%)
Rural	568 (1.2%)	887 (1.8%)	12,530 (3.4%)	19,718 (4.8%)	246 (0.8%)	1,391 (2.2%)	2,031 (3.3%)

There was a marked decline in the prevalence of thinness between 2006 and 2016 (absolute change: women − 8.0%; men − 9.0%). The prevalence of overweight also increased consistently between 2006 and 2016 (absolute change: women + 2.7%; men + 5.4%) whereas the prevalence of obesity rose most rapidly between 2016 and 2021 (absolute change: women + 1.2%; men + 1.2%) (Table 2; Figs. 1 and 2).

Rural areas consistently show a higher rate of thinness compared to urban areas, which exhibit greater instances of overweight and obesity. For instance, the rate of severe or moderate thinness in urban India in 2021 was 3.5% for women and 2.5% for men, whereas in rural regions, these figures rose to 6.2% and 3.5%, respectively. Conversely, obesity rates were higher in urban settings, with 11.0% of women and 6.6% of men being affected in 2021, compared to 4.8% of women and 3.3% of men in rural locations. The patterns of BMI change for rural and urban populations followed the overall trend of decreasing thinness and increasing overweight/obesity. (Table 2, Figure S1 , Figure S2).

More than half of the population consistently were classified as “Normal” BMI, ranging from 55 to 65% **(**Table 2**)**.

### Changes in the geographic distribution of BMI categories

In 2021, the states with the highest obesity prevalence were Puducherry (women: 20.2%, men: 10.1%), Chandigarh (women: 19.0%, men: 10.0%) and Delhi (women: 16.4%, men: 7.8%). These states also had a high prevalence of overweight. Dadra and Nagar Haveli and Diu (women: 9.8%, men: 6.0%), Gujarat (women: 9.3%, men: 6.6%), Jharkhand (women: 8.0%, men: 3.4%), and Bihar (women: 8.0%, men: 4.4%) had high severely/moderately thin populations. Distributions of mildly thin are similar (Figure S3). The standard deviation of Thin across states decreased from 1999 to 2021, while it increased for obesity (Fig. 2).

**Fig. 3:**
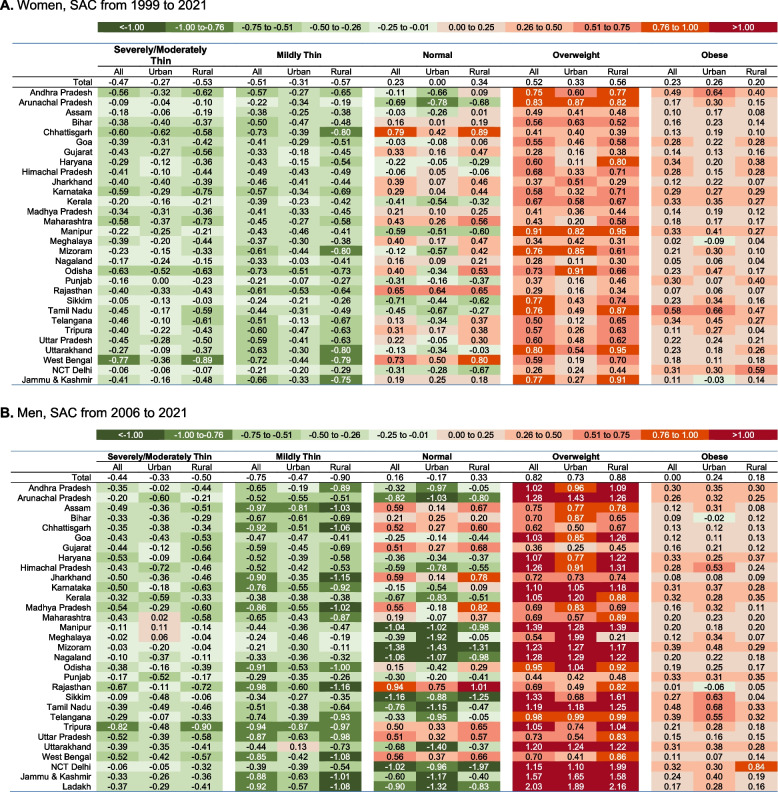
Standardized Absolute Change (SAC, percentage points) during study period in the prevalence of Body Mass Index (BMI) categories across States/Union Territories. **A** Women, SAC from 1999 to 2021. The states/union territories of Ladakh, Andaman & Nicobar, Chandigarh, Dadra and Nagar Haveli and Daman and Diu, Lakshadweep, and Puducherry were excluded from the SAC calculation as these regions were not included in the NFHS survey in 1999. **B** Men, SAC from 2006 to 2021. The states/union territories of Andaman & Nicobar, Chandigarh, Dadra and Nagar Haveli and Daman and Diu, Lakshadweep, and Puducherry were excluded from the SAC calculation as these regions were not included in the NFHS survey in 2006

For women, all states witnessed a decline in severely/moderately thin populations, with West Bengal showing the largest decrease of 0.77% points annually over 22 years between 1999 and 2021. Thin BMI populations steadily decreased, but some states (Assam, Bihar, Madhya Pradesh, and Tripura) showed an increase between 1999 and 2006. Obesity substantially increased in Tamil Nadu (SAC 1999–2021: 0.58%), Andhra Pradesh (0.49%), and Haryana (0.34%) (Fig. 3, Figure S4). Figure S4 shows the detailed SAC between each survey period.

Trends for men were similar to those in women, but the prevalence of extreme categories (severely/moderately thin and obese) was less than for women. Tripura (-0.82%) and Rajasthan (-0.67%) showed the most prominent decrease in severely/moderately thin populations between 2006 and 2021. (Fig. 3, Figure S4). Obesity in men increased most in the states of Tamil Nadu (+ 0.48%) and Telangana (+ 0.39%) across 2006 and 2021.

Rural populations have undergone more significant changes in weight distribution than their urban counterparts across various states. This contrast is evident when comparing the heat maps of urban and rural India in Fig. 3. Specifically, urban populations saw an annual increase in overweight prevalence of 0.33% points for women and 0.73% points for men during the study period. Meanwhile, rural populations experienced a more pronounced annual increase, with 0.56% points for women and 0.88% points for men in overweight prevalence.

To understand how the rank-ordering of BMI outcomes has changed over time, we calculated a correlation between the rankings across years. A correlation close to 1 indicates the ranking of states has not changed much over time and a smaller value suggests changes in the ranking (Table S2). The rank correlation between the ordering of states/UTs was strong (> 0.7) for all outcomes except normal BMI for both women and men.

### Estimated headcount of BMI outcomes in India

**Fig. 4:**
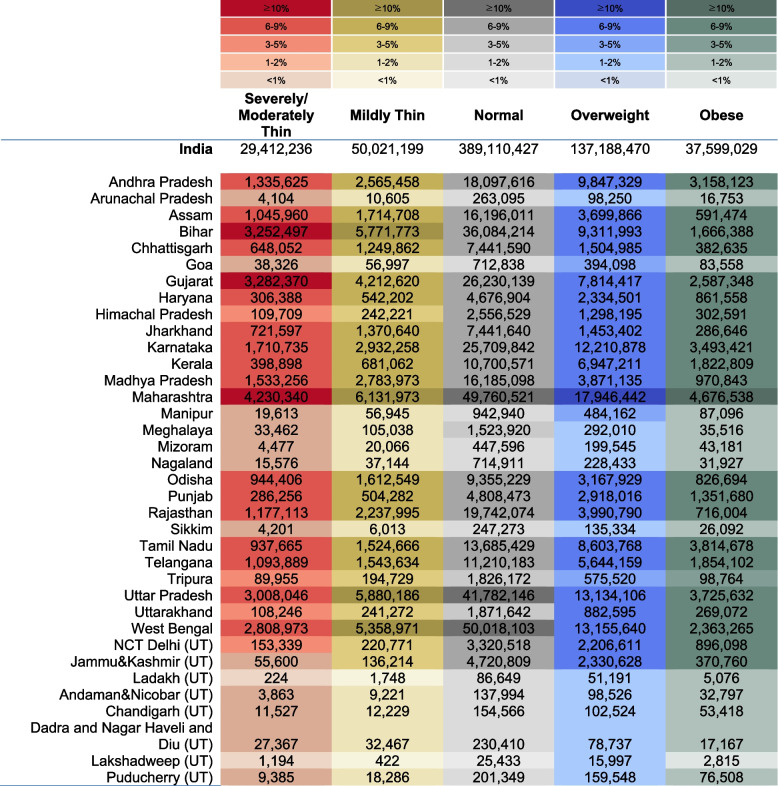
Estimated headcounts of BMI categories for India and 36 States/Union Territories, 2021. Headcounts were calculated for men and women separately and summed up to estimate values for the entire population. Colors indicate the share of each state in India's total burden, with darker shades representing a larger proportion

In 2021, approximately 29,412,236 adults were severely/moderately thin in India (Fig. 4). The population headcount varied from 4,230,340 in Maharashtra to 224 in Ladakh. Maharashtra (13.94%), Gujarat (10.82%), Bihar (10.72%) and Uttar Pradesh (9.91%) account for 45.39% of the total burden of severely/moderately thin in India.

In 2021, approximately 37,599,029 adults were obese in India (Fig. 4). The population headcount varied from 4,676,538 in Maharashtra to 2,815 in Lakshadweep. Maharashtra (12.05%), Tamil Nadu (9.83%), Uttar Pradesh (9.60%), and Karnataka (9.00%) account for 40.48% of the total burden of obesity in India.

**Fig. 5:**
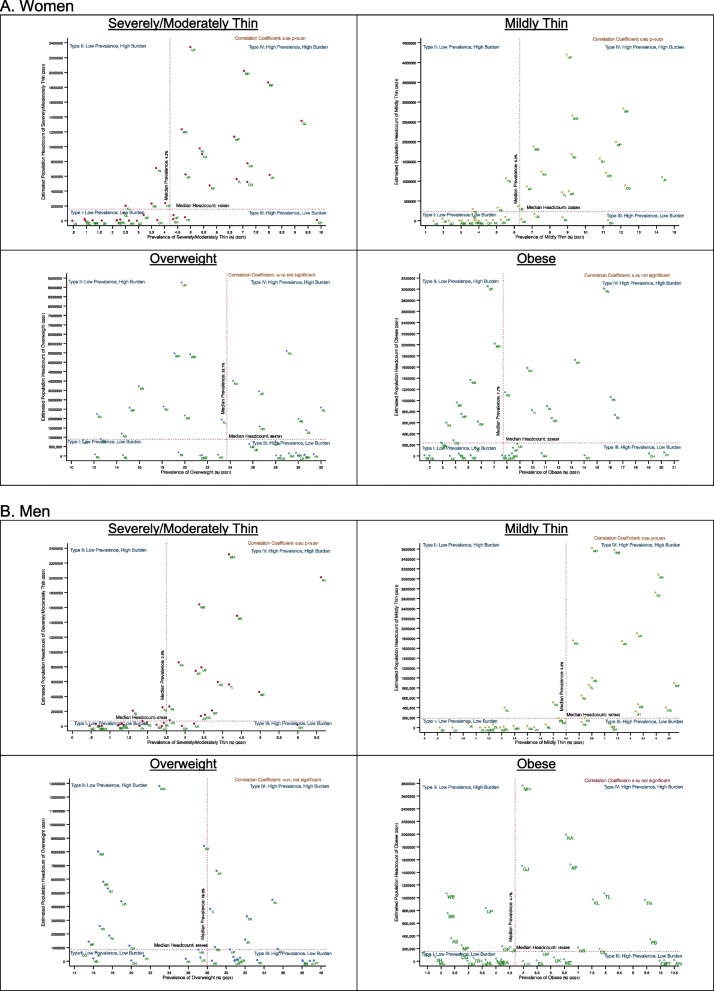
Relationship between the prevalence and Headcount Burden of BMI categories, 2021. AN: Andaman & Nicobar, AP: Andhra Pradesh, AR: Arunachal Pradesh, AS: Assam, BR: Bihar, CH: Chandigarh, CG: Chhattisgarh, DH: Dadra and Nagar Haveli and Daman and Diu, GA: Goa, GJ: Gujarat, HR: Haryana, HP: Himachal Pradesh, JK: Jammu & Kashmir, JH: Jharkhand, KA: Karnataka, KL: Kerala, LK: Ladakh, LD: Lakshadweep, MP: Madhya Pradesh, MH: Maharashtra, MN: Manipur, ML: Meghalaya, MZ: Mizoram, DL: NCT Delhi, NL: Nagaland, OR: Odisha, PY: Puducherry, PB: Punjab, RJ: Rajasthan, SK: Sikkim, TN: Tamil Nadu, TL: Telangana, TR: Tripura, UP: Uttar Pradesh, UK: Uttarakhand, WB: West Bengal

States/UTs with a higher prevalence of thinness, on average, tend to have a larger absolute burden for both men and women (severely/moderately thin: women *r* = 0.59, men *r* = 0.60; mildly thin: women *r* = 0.60, men *r* = 0.55) (Fig. 5). Distributions of states for severely/moderately thin and mildly thin resemble each other. Andhra Pradesh, Assam, Bihar, Chhattisgarh, Gujarat, Jharkhand, Karnataka, Madhya Pradesh, Maharashtra, Odisha, Telangana, Uttar Pradesh, and West Bengal are states with High Prevalence and High Burden (headcount) (Type IV) for both severely/moderately thin and mildly thin in case of both men and women. Himachal Pradesh and Tripura have Low Prevalence and Low Burden (Type I).

On the other hand, the relationship between headcount burden and prevalence of overweight and obese is not as clear as thin populations. The distribution is similar for overweight and obesity for both genders. Tamil Nadu, Andhra Pradesh, Kerala, Punjab, and Delhi are states with High Prevalence and High Burden (headcount) (Type IV) of overweight and obesity. Meghalaya, Nagaland, and Tripura have Low Prevalence and Low Burden (Type I). The relationship for normal BMI is shown in Figure S5.

## Discussion

To the best of our knowledge, there was hardly any study that analyzed BMI trends in India over 20 years including the most recent data of NFHS (2019–2021). The study has 5 salient findings. First, the prevalence of thinness is decreasing, whereas overweight/obesity rates are rising over the years. Second, we found differences in the trends in men and women. Women have a larger prevalence of extreme categories (severely/moderately thin and obese) than men. Third, while rural populations exhibit a higher prevalence of thinness compared to urban populations, they are undergoing a faster change in BMI distribution than their urban counterparts. Fourth, the boxplots and the strong correlation in prevalence rankings across periods suggest a lasting state-level inequality of malnutrition. Fifth, the biggest headcounts of severely/moderately thin are found in Maharashtra, Gujarat, Bihar, and Uttar Pradesh, which together account for 45% of the total burden. This may indicate the combined effects of the states’ population sizes and their respective rates of under- and over-nutrition. Similarly, the greatest burden of obesity is concentrated in Maharashtra, Tamil Nadu, Uttar Pradesh, and Karnataka, representing 40% of the total burden.

The primary strength of this study lies in its use of extensive, nationally representative data, making the findings applicable at the national level. Additionally, while much of the existing literature has primarily concentrated on the correlates of BMI among women of reproductive age, our study offers insights into the prevalence of both underweight and overweight/obesity conditions across Indian states for adult men and women.

There were several limitations of the study. First, the absence of data on men in the NFHS-2 survey (1998-99) complicates the gender comparison over the years. Second, the NFHS-5, initiated in 2019, encountered disruptions due to the COVID-19 pandemic, which impacted the continuity and comprehensiveness of the data collection process [27]. Third, the upper limit of age was limited to 49 for women and 54 for men in the surveys, which restricts the ability to generalize the study’s findings to the changes affecting older population groups.

The variation in BMI distribution across different states can be attributed to a multitude of factors, such as distinct demographics, socioeconomic conditions, cultural norms, languages, geographical landscapes, and the specific governance and policy frameworks of each state. Notably, the Empowered Action Group (EAG) States, which include Rajasthan, Uttar Pradesh, Uttarakhand, Bihar, Jharkhand, Madhya Pradesh, Chhattisgarh, and Odisha, exhibit a higher prevalence of underweight individuals. This trend underscores the socioeconomic challenges these states face, as evidenced by their lagging demographic and social indicators. This situation calls for focused policy interventions, especially since more than 5% of women in six out of these eight states were found to be severely or moderately underweight. Meanwhile, the recently merged union territories of Dadra and Nagar Haveli and Diu also showed significant thinness among both genders. In contrast, the southern states of Andhra Pradesh, Karnataka, Kerala, Tamil Nadu, and Telangana reported higher rates of overweight and obesity in 2021, continuing the trend observed in 2016, as per recent studies [19]. 

The findings suggest that the DBM persists in India. The distribution of thinness remains largely unchanged across states, showing a high clustering in most of the Empowered Action Group States. With rapidly increasing overweight and obesity, DBM in India calls for attention. The proposal by the WHO and the United Nations could help devise proactive measures to prevent DBM; for example, building food systems for healthy, sustainable diets; social protection and nutrition-related education; and healthcare strengthening for providing universal coverage of essential nutrition actions [37]. 

Additionally, gender differences in BMI are notable. The gender differences in overweight/obesity in India can be attributed to health risk factors, such as lower physical activity among women respondents [38]. Postpartum weight retention could also cause higher reported overweight/obesity for women than in men [39]. Other contributing elements might include varying social pressures and environmental factors that influence health behaviors associated with BMI.

The reasons for the more rapid change in BMI distribution among rural populations, particularly in thinness, are unclear. The NFHS defines rural areas as those not classified as urban. Urban areas encompass statutory towns, determined by administrative bodies, and census towns, defined by population size and density [40]. Consequently, the classification of what constitutes a rural area may have changed over the study period, thus complicating the interpretation of results. Future studies should investigate the various societal influences on BMI distribution across different regions in India.

Examining the causes behind ongoing state-level variations in underweight prevalence and rising obesity is vital for developing public health strategies to address these challenges. Socioeconomic factors, including income and education [41], impact BMI differently across states and social classes, highlighting the need for in-depth analysis to fully understand these dynamics. Addressing the enduring disparities in underweight and obesity rates across states requires more nuanced policy actions. Research should focus on pinpointing specific regions, like districts, sub-districts, and villages, and targeting populations vulnerable due to their sociodemographic characteristics to enable more effective interventions.

## Conclusions

The prevalence of thinness is consistently decreasing while the prevalence of overweight/obesity has been increasing over the years. Prevalence of extreme categories (severely/moderately thin and obese) was larger in the case of women than men. We have found evidence of persisting inequality between states, especially for undernutrition outcomes. Tailored efforts at state levels are required to further strengthen existing policies and develop new interventions to target both forms of malnutrition.

### Supplementary Information

Supplementary Material 1.

## Data Availability

No datasets were generated or analysed during the current study.

## Abbreviations

BMI: Body mass index
NFHS: National family health survey
UT: Union territories
SDG: Sustainable development goal
LMIC: Low- and middle- income countries
DBM: Double burden of malnutrition
WHO: World health organization
SAC: Standardized absolute change
IPUMS: Integrated public use microdata series
EAG: Empowered action group states
