# Supplementary material for: Temporal change in prevalence of BMI categories in India: patterns across States and Union territories of India, 1999–2021
Source: BMC Public Health. 2024 May 16;24:1322. doi: 10.1186/s12889-024-18784-4 (PMC11097587; doi:10.1186/s12889-024-18784-4)
Supplement: Supplementary file 1 — Supplementary Material 1. [file 12889_2024_18784_MOESM1_ESM.docx]

**Temporal change in prevalence of BMI categories in India:**

**Patterns across States and Union Territories of India, 1999-2021**

**Supplementary Materials**

Sung M, Kumar A, Mishra R, Kulkarni B, Kim R, Subramanian S V. Change in prevalence of BMI categories in India: Patterns across States and Union Territories of India, 1999-2021.

**Appendix S1: Studies found from a systematic search and reviewed as a part of our study**

**Evidence before the study**

We searched PubMed and Web of Science to identify empirical studies with a quantitative analysis on the trends of BMI distribution in India. We used structured combinations of keywords: ("BMI" OR "body mass index" OR "Underweight" OR "Overweight" OR "Obese" OR "Thin" OR "Chronic Energy Deficit") AND (" Adults" OR "Women" OR "Men" ) AND ("Trends" OR "Change" OR "Time") AND ("India") with no restriction on date and language. The search yielded 1035 studies. After removing duplicates and screening the titles and abstracts of each of these papers, 47 articles were considered as relevant for the scope of our study. We reviewed these articles for the following: focus of the study, year range of study, and source of data. Four studies focused on chronic energy deficiency or undernutrition, twenty-four studies focused on overweight or obese, and seventeen literatures studied the general change of body mass index distributions. Eight studies emphasized the double burden of malnutrition of lower socioeconomic groups. Majority of the studies (n=33) used data from the National Family Health Survey (NFHS) from 1998-1999 (NFHS-2), 2005-2006 (NFHS-3), and 2015-2016 (NFHS-4). However, none of these studied reviewed BMI trends including the most recent data of 2019-2021(NFHS-5) or analyzed distribution over 20 years using public data set.

**Bibliography of the studies**

1. Agrawal, A., C. Lalji, and D. Pakrashi, *He Has Gone to a Better Place, but She Has Not: Health Status of Hindu Widows in India.* Journal of Development Studies, 2021. **57**(5): p. 750-771.

2. Agrawal, P., et al., *Effects of Sedentary Lifestyle and Dietary Habits on Body Mass Index Change among Adult Women in India: Findings from a Follow-Up Study.* Ecology of Food and Nutrition, 2013. **52**(5): p. 387-406.

3. Al Kibria, G.M., et al., *Prevalence and factors associated with underweight, overweight and obesity among women of reproductive age in India.* Global Health Research and Policy, 2019. **4**(1).

4. Alvarez-Saavedra, V., P. Levasseur, and S. Seetahul, *The Role of Gender Inequality in the Obesity Epidemic: A Case Study from India.* Journal of Development Studies, 2023. **59**(7): p. 980-996.

5. Balarajan, Y. and E. Villamor, *Nationally Representative Surveys Show Recent Increases in the Prevalence of Overweight and Obesity among Women of Reproductive Age in Bangladesh, Nepal, and India.* Journal of Nutrition, 2009. **139**(11): p. 2139-2144.

6. Bhandari, P., E. Gayawan, and S. Yadav, *Double burden of underweight and overweight among Indian adults: spatial patterns and social determinants.* Public Health Nutr, 2021. **24**(10): p. 2808-2822.

7. Chaurasiya, D., et al., *Age, period and birth cohort effects on prevalence of obesity among reproductive-age women in India.* Ssm-Population Health, 2019. **9**.

8. Das, P., T. Das, and T.B. Roy, *Overweight Syndrome, a Deviation from Normalcy: a Multiple Risk Factor Analysis Among Reproductive Age Group (15-49) Women in Indian Context.* Global Social Welfare, 2022. **9**(3): p. 157-167.

9. Dutta, M., et al., *The double burden of malnutrition among adults in India: evidence from the National Family Health Survey-4 (2015-16).* Epidemiol Health, 2019. **41**: p. e2019050.

10. Ebrahim, S., et al., *The Effect of Rural-to-Urban Migration on Obesity and Diabetes in India: A Cross-Sectional Study.* Plos Medicine, 2010. **7**(4).

11. Gouda, J. and R.K. Prusty, *Overweight and Obesity among Women by Economic Stratum in Urban India.* Journal of Health Population and Nutrition, 2014. **32**(1): p. 79-88.

12. Hadaye, R.S., R.M. Manapurath, and B.P. Gadapani, *Obesity prevalence and determinants among young adults, with special focus on normal-weight obesity; a cross-sectional study in Mumbai.* Indian Journal of Community Medicine, 2020. **45**(3): p. 358-362.

13. Kim, R., et al., *Heterogeneity in adult anthropometry by socioeconomic factors: Indian National Family Health Survey 2006 and 2016.* European Journal of Clinical Nutrition, 2020. **74**(6): p. 953-960.

14. Kulkarni, V.S., V.S. Kulkarni, and R. Gaiha, *"Double Burden of Malnutrition": Reexamining the Coexistence of Undernutrition and Overweight Among Women in India.* International Journal of Health Services, 2017. **47**(1): p. 108-133.

15. Kumar, P., et al., *Prevalence and factors associated with triple burden of malnutrition among mother-child pairs in India: a study based on National Family Health Survey 2015-16.* Bmc Public Health, 2021. **21**(1).

16. Kumar, P., S. Mangla, and S. Kundu, *Inequalities in overweight and obesity among reproductive age group women in India: evidence from National Family Health Survey (2015–16).* BMC Women's Health, 2022. **22**(1): p. 205.

17. Liczbinska, G., et al., *Body size and weight status of adult Indian males born in the 1890s-1950s: Age and secular change in the context of demographic, economic, and political transformation.* American Journal of Human Biology, 2023.

18. Little, M., et al., *Socio-demographic patterning of the individual-level double burden of malnutrition in a rural population in South India: a cross-sectional study.* Bmc Public Health, 2020. **20**(1).

19. Little, M., et al., *Factors associated with BMI, underweight, overweight, and obesity among adults in a population of rural south India: a cross-sectional study.* BMC Obesity, 2016. **3**(1): p. 12.

20. Luhar, S., et al., *Trends in the socioeconomic patterning of overweight/obesity in India: a repeated cross-sectional study using nationally representative data.* Bmj Open, 2018. **8**(10).

21. Luhar, S., et al., *Do trends in the prevalence of overweight by socio-economic position differ between India's most and least economically developed states?* Bmc Public Health, 2019. **19**.

22. Moli, G.K. and G.K. Mini, *Correlates of Overweight among Adult Women in Kerala: A Study Based on National Family Health Survey (NFHS-3).* Anthropologist, 2012. **14**(1): p. 67-72.

23. Mungreiphy, N.K. and S. Kapoor, *SOCIOECONOMIC CHANGES AS COVARIATES OF OVERWEIGHT AND OBESITY AMONG TANGKHUL NAGA TRIBAL WOMEN OF MANIPUR, NORTH-EAST INDIA.* Journal of Biosocial Science, 2010. **42**(3): p. 289-305.

24. Nguyen, P.H., et al., *The double burden of malnutrition in India: Trends and inequalities (2006-2016).* Plos One, 2021. **16**(2).

25. Patel, M.L. and R. Deonandan, *Factors associated with body mass index among slum dwelling women in India: an analysis of the 2005–2006 Indian National Family Health Survey.* International Journal of General Medicine, 2017. **10**: p. 27-31.

26. Patel, R., et al., *Factors associated with double burden of malnutrition among mother-child pairs in India: A study based on National Family Health Survey 2015-16.* Children and Youth Services Review, 2020. **116**.

27. Pengpid, S. and K. Peltzer, *Prevalence and correlates of underweight and overweight/obesity among women in India: results from the National Family Health Survey 2015-2016.* Diabetes Metabolic Syndrome and Obesity-Target & Therapy, 2019. **12**: p. 647-653.

28. Rai, R.K., *Factors Associated With Nutritional Status Among Adult Women in Urban India, 1998-2006.* Asia-Pacific Journal of Public Health, 2015. **27**(2): p. NP1241-NP1252.

29. Rai, R.K., et al., *Prospective cohort study of overweight and obesity among rural Indian adults: sociodemographic predictors of prevalence, incidence and remission.* Bmj Open, 2018. **8**(8).

30. Rai, R.K., et al., *Rising burden of overweight and obesity among Indian adults: empirical insights for public health preparedness.* Journal of Biosocial Science, 2021. **53**(5): p. 709-723.

31. Razak, F., et al., *Prevalence of Body Mass Index Lower Than 16 Among Women in Low- and Middle-Income Countries.* Jama-Journal of the American Medical Association, 2015. **314**(20): p. 2164-2171.

32. Sauvaget, C., et al., *Body mass index, weight change and mortality risk in a prospective study in India.* International Journal of Epidemiology, 2008. **37**(5): p. 990-1004.

33. Sengupta, A., et al., *Overweight and obesity prevalence among Indian women by place of residence and socio-economic status: Contrasting patterns from 'underweight states' and 'overweight states' of India.* Social Science & Medicine, 2015. **138**: p. 161-169.

34. Sengupta, A., et al., *State-wise Dynamics of the Double Burden of Malnutrition among 15-49 Year-old Women in India: How Much Does the Scenario Change Considering Asian Population-specific BMI Cut-off Values?* Ecology of Food and Nutrition, 2014. **53**(6): p. 618-638.

35. Shannawaz, M. and P. Arokiasamy, *Overweight/Obesity: An Emerging Epidemic in India.* Journal of Clinical and Diagnostic Research, 2018. **12**(11): p. LC1-LC5.

36. Shirisha, P., V.R. Muraleedharan, and G. Vaidyanathan, *Wealth related inequality in women and children malnutrition in the state of Chhattisgarh and Tamil Nadu.* Bmc Nutrition, 2022. **8**(1).

37. Siddiqui, Z. and R. Donato, *The dramatic rise in the prevalence of overweight and obesity in India: Obesity transition and the looming health care crisis.* World Development, 2020. **134**.

38. Singh, G., et al., *Overweight and obesity, the clock ticking in India? A secondary analysis of trends of prevalence, patterns, and predictors from 2005 to 2020 using the National Family Health Survey.* International Journal of Noncommunicable Diseases, 2023. **8**(1): p. 31-45.

39. Singh, M.S. and J.W. Dkhar, *Growing Obese in Manipur: Relationship between Age and Obesity among the Meitei Males of Manipur, India.* Anthropologist, 2013. **16**(3): p. 753-756.

40. Subramanian, S.V., J.M. Perkins, and K.T. Khan, *Do burdens of underweight and overweight coexist among lower socioeconomic groups in India?* American Journal of Clinical Nutrition, 2009. **90**(2): p. 369-376.

41. Subramanian, S.V. and G.D. Smith, *Patterns, distribution, and determinants of under- and overnutrition: a population-based study of women in India2.* The American Journal of Clinical Nutrition, 2006. **84**(3): p. 633-640.

42. Swain, S. and S. Chowdhury, *Trends of nutritional status among rural adults in six states of India: findings from national survey data.* Clinical Epidemiology and Global Health, 2018. **6**(4): p. 181-187.

43. Tandon, K., S. Kapoor, and A.K. Kapoor, *Covariates and Prevalence of Obesity among Adult North Indian Population.* Collegium Antropologicum, 2011. **35**(2): p. 305-311.

44. Verma, M., et al., *Epidemiology of overweight and obesity in Indian adults - A data of the National Health.* Diabetes & Metabolic Syndrome-Clinical Research & Reviews, 2021. **15**(4).

45. Wang, Y., et al., *Is obesity becoming a public health problem in India? Examine the shift from under- to overnutrition problems over time.* Obes Rev, 2009. **10**(4): p. 456-74.

46. Yaya, S. and B. Ghose, *Change in nutritional status among women of childbearing age in India (1998-2016).* Obesity Science & Practice, 2020. **6**(5): p. 535-543.

47. Young, M.F., et al., *A Double Edged Sword? Improvements in Economic Conditions over a Decade in India Led to Declines in Undernutrition as Well as Increases in Overweight among Adolescents and Women.* Journal of Nutrition, 2020. **150**(2): p. 364-372.

**Appendix S2**: National Family Health Survey (NFHS): Summary of Survey Design

**NFHS-2 (1998-1999) and NFHS-3 (2005-2006)**

The NFHS-2 sample covers more than 99 percent of India’s population living in all 26 states. It does not cover the union territories. NFHS-2 is a household survey with an overall target sample size of approximately 90,000 ever-married women in the age group 15–49. The target sample size was set considering the size of the state, the resources available for the survey, and the aggregate level (urban/rural, region, metropolitan cities) at which separate estimates were needed. The initial target sample size was 4,000 completed interviews with eligible women in states with a 1991 population of more than 25 million, 3,000 completed interviews with eligible women in states with a 1991 population between 2 and 25 million, and 1,500 completed interviews with eligible women in states with a population of less than 2 million. A uniform sample design was adopted in all the states. In each state, the rural sample was selected in two stages: the selection of Primary Sampling Units (PSUs), which are villages, with probability proportional to population size (PPS) at the first stage, followed by the random selection of 9 households within each PSU in the second stage. In urban areas, a three-stage procedure was followed. In the first stage, wards were selected with PPS sampling. In the next stage, one census enumeration block (CEB) was randomly selected from each sample ward (except in Jammu and Kashmir, where two CEBs were randomly selected from each sample ward). In the final stage, households were randomly selected within each sample CEB. In NFHS 3, the sampling frame was census 2001 data.

**NFHS-4 (2015-2016) and NFHS-5 (2019-2021)**

NFHS-4 and NFHS-5 are designed to provide estimates at district levels. The sample is a stratified two-stage sample. The 2011 census served as the sampling frame for the selection of PSUs. PSUs were villages in rural areas and Census Enumeration Blocks (CEBs) in urban areas. PSUs with fewer than 40 households were linked to the nearest PSU. Within each rural stratum, villages were selected from the sampling frame with probability proportional to size (PPS). In each stratum, six approximately equal substrata were created by crossing three substrata, each created based on the estimated number of households in each village, with two substrata, each created based on the percentage of the population belonging to scheduled castes and scheduled tribes (SCs/STs). Within each explicit sampling stratum, PSUs were sorted according to the literacy rate of women age 6+ years. The final sample PSUs were selected with PPS sampling. In urban areas, CEB information was obtained from the Office of the Registrar General and Census Commissioner, New Delhi. CEBs were sorted according to the percentage of the SC/ST population in each CEB, and sample CEBs were selected with PPS sampling. In every selected rural and urban PSU, a complete household mapping and listing operation was conducted prior to the main survey. Selected PSUs with an estimated number of at least 300 households were segmented into segments of approximately 100-150 households. Two of the segments were randomly selected for the survey using systematic sampling with probability proportional to segment size. Therefore, an NFHS-4 cluster is either a PSU or a segment of a PSU. In the second stage, in every selected rural and urban cluster, 22 households were randomly selected with systematic sampling.

**Appendix S3**: Linking data from all five National Family Health Surveys (NFHS) to the 2021 (latest) States and Union Territories

This method relies on assigning all clusters within a particular district to a new state that was formed from a parent state that was surveyed in earlier NFHS surveys.

NFHS-2 surveyed 440 districts in 26 states (at time of the survey) and out of the 440 districts, all of them were identified by IPUMS. This identification was possible because Demographic and Health Surveys (DHS) released district codes in NFHS-2 that matched the district’s sampling frame codes. In NFHS-3, DHS implemented a scrambling process that prevented the identification of districts. Both NFHS-4 and NFHS-5 were district representative surveys and therefore had publicly available district codes. We used the methods of a recent paper”† to track the formation of new states and districts.

The NFHS surveys were not designed with the intention that the states would be broken apart in the future, and because of this the results generated from this method may not be fully state representative. The summary of how the state equivalence was created for each NFHS survey is outlined in the Table below:

Summary of the Method Used for Creating State Equivalence for each NFHS Survey

| **NFHS Survey and Time Period** | **State Equivalence Method Summary** |
| --- | --- |
| NFHS-2: 1998-1999 | Districts were re-assigned to the current State. The results are presented in table below. |
| NFHS-3: 2005-2006 | No district data is available, so pre-existing state value is applied to the parent and new state when a new state was formed. |
| NFHS-4: 2015-2016 | Kargil and Leh districts from Jammu and Kashmir were assigned to the new union territory of Ladakh. Dadra and Nagar Haveli and Daman and Diu were merged into one state. |
| NFHS-5: 2019-2021 | No changes were made. |

**References**

†Subramanian SV, Ambade M, Sharma S, Kumar A, Kim R. Prevalence of Zero-Food among infants and young children in India: patterns of change across the States and Union Territories of India, 1993-2021. EClinicalMedicine. 2023 Mar 30;58:101890. doi: 10.1016/j.eclinm.2023.101890. Erratum in: EClinicalMedicine. 2023 Jun 27;61:102047. PMID: 37065175; PMCID: PMC10102207.

We have outlined whether there is state representative data for each NFHS survey in the following:

Legend shows the Data Availability Categories Used in this Method:

| ✓ = State Representative Data Available | ⚠ = Not State Representative 🡪 Districts were Re-Assigned | 🗶 = No Data Available |
| --- | --- | --- |

Overview of Data from each NFHS Survey for each State or Union Territory

| **States and Union Territories (2021)** | **NFHS-2**  **1998-1999** | **NFHS-3**  **2005-2006** | **NFHS-4**  **2015-2016** | **NFHS-5**  **2019-2021** |
| --- | --- | --- | --- | --- |
| Andhra Pradesh | ⚠ | ⚠ | ✓ | ✓ |
| Arunachal Pradesh | ✓ | ✓ | ✓ | ✓ |
| Assam | ✓ | ✓ | ✓ | ✓ |
| Bihar | ⚠ | ✓ | ✓ | ✓ |
| Chhattisgarh | ⚠ | ✓ | ✓ | ✓ |
| Goa | ✓ | ✓ | ✓ | ✓ |
| Gujarat | ✓ | ✓ | ✓ | ✓ |
| Haryana | ✓ | ✓ | ✓ | ✓ |
| Himachal Pradesh | ✓ | ✓ | ✓ | ✓ |
| Jharkhand | ⚠ | ✓ | ✓ | ✓ |
| Karnataka | ✓ | ✓ | ✓ | ✓ |
| Kerala | ✓ | ✓ | ✓ | ✓ |
| Madhya Pradesh | ⚠ | ✓ | ✓ | ✓ |
| Maharashtra | ✓ | ✓ | ✓ | ✓ |
| Manipur | ✓ | ✓ | ✓ | ✓ |
| Meghalaya | ✓ | ✓ | ✓ | ✓ |
| Mizoram | ✓ | ✓ | ✓ | ✓ |
| Nagaland | ✓ | ✓ | ✓ | ✓ |
| Odisha | ✓ | ✓ | ✓ | ✓ |
| Punjab | ✓ | ✓ | ✓ | ✓ |
| Rajasthan | ✓ | ✓ | ✓ | ✓ |
| Sikkim | ✓ | ✓ | ✓ | ✓ |
| Tamil Nadu | ✓ | ✓ | ✓ | ✓ |
| Telangana | ⚠ | ⚠ | ✓ | ✓ |
| Tripura | ✓ | ✓ | ✓ | ✓ |
| Uttar Pradesh | ⚠ | ✓ | ✓ | ✓ |
| Uttarakhand | ⚠ | ✓ | ✓ | ✓ |
| West Bengal | ✓ | ✓ | ✓ | ✓ |
| Andaman & Nicobar Island (UT) | 🗶 | 🗶 | ✓ | ✓ |
| Chandigarh (UT) | 🗶 | 🗶 | ✓ | ✓ |
| Dadra Nagar Haveli & Daman & Diu (UT) | 🗶 | 🗶 | ✓ | ✓ |
| NCT Delhi (UT) | ✓ | ✓ | ✓ | ✓ |
| Jammu & Kashmir (UT) | ⚠ | ⚠ | ✓ | ✓ |
| Ladakh (UT) | 🗶 | ⚠ | ✓ | ✓ |
| Lakshadweep (UT) | 🗶 | 🗶 | ✓ | ✓ |
| Puducherry (UT) | 🗶 | 🗶 | ✓ | ✓ |

†These marked states are not state representative as they had districts added or removed from them.

For NFHS-2, refer below to see the number of districts at the time of each survey that were assigned to the new states. States marked with † are not state representative and either had districts removed or added to them.

Number of Districts from NFHS-1 and NFHS-2 that fall into the current states

| **States and Union Territories (2021)** | **NFHS-2** |
| --- | --- |
| Andhra Pradesh† | 13 |
| Arunachal Pradesh | 11 |
| Assam | 20 |
| Bihar† | 29 |
| Chhattisgarh† | 7 |
| Goa | 2 |
| Gujarat | 19 |
| Haryana | 16 |
| Himachal Pradesh | 11 |
| Jharkhand† | 12 |
| Karnataka | 20 |
| Kerala | 14 |
| Madhya Pradesh† | 37 |
| Maharashtra | 28 |
| Manipur | 8 |
| Meghalaya | 5 |
| Mizoram | 3 |
| Nagaland | 7 |
| Odisha | 13 |
| Punjab | 12 |
| Rajasthan | 27 |
| Sikkim | 4 |
| Tamil Nadu | 21 |
| Telangana† | 10 |
| Tripura | 3 |
| Uttar Pradesh† | 53 |
| Uttarakhand† | 8 |
| West Bengal | 17 |
| Andaman & Nicobar Island (UT) | 0 |
| Chandigarh (UT) | 0 |
| Dadra Nagar Haveli & Daman & Diu (UT) | 0 |
| NCT Delhi (UT) | 1 |
| Jammu & Kashmir (UT) † | 9 |
| Ladakh (UT) † | 0 |
| Lakshadweep (UT) | 0 |
| Puducherry (UT) | 0 |
| **Districts not Assigned to any State** | 0 |

†These marked states are not state representative as they had districts added or removed from them.

**Appendix S4**: Methodology used for calculating the population headcount of BMI outcomes

To calculate the headcount, we followed IPUMS POPWT methodology^1^ with a few adjustments to the formulas. To derive an estimated all-India adult population for 2021, we used the Census 2011 Population Projections published in July 2020.^2^

| **Gender** | **Total Population (Thousands)** | **Method Used** | **Source** |
| --- | --- | --- | --- |
| Women | 303,445 | Took the population ranging 20-49 years from Table 18 (2021). Excluded the proportion of women who is pregnant or recently gave birth in the last two months from the NFHS5 survey. | Census 2011 Projections |
| Men | 356,244 | Took the population ranging 20-54 years from Table 18 (2021) | Census 2011 Projections |

After deriving the men and women population for all-India, this paper used the formula given by IPUMS to derive the population weight (POPWT) which was used to calculate the headcount.

$$popwt=\left( \frac{wt}{sum\left( wt \right)} \right)\times Pop$$

- “*wt*” represents the individual weight divided by 1,000,000 in the DHS microdata.
- “*sum(wt)*” represents the total value of wt
- “*Pop*” represents the total all-India estimated population of women/men.

Weighting the data using popwt instead of the given individual weights, this paper was able to total headcounts of each BMI outcomes for all-India and its states/union territories.

**References**

1. IPUMS DHS. POPWT Expansion factor. September 9, 2022. Available from: <https://www.idhsdata.org/idhs/population_weights.shtml>
2. National Commission on Population, Ministry of Health and Family Welfare, Government of India. Population Projections for India and States 2011-2036: Report of the Technical Group on Population Projections. 2020: Available from:<https://main.mohfw.gov.in/sites/default/files/Population%20Projection%20Report%202011-2036%20-%20upload_compressed_0.pdf>

**<Supplementary Exhibits>**

**Table S1.** Women sample size and BMI prevalence comparison using Individual Recode dataset versus Household Member Recode dataset

|  | **NFHS5 (2021)** | | **NFHS4 (2016)** | | **NFHS3 (2006)** | |
| --- | --- | --- | --- | --- | --- | --- |
|  | IR* | PR** | IR | PR | IR | PR |
| Total sample (n) | 90,333 | 93,436 | 531,433 | 540,980 | 551,027 | 559,098 |
| **BMI category** |  |  |  |  |  |  |
| **Severely/Moderately Thin** |  |  |  |  |  |  |
| All | 10,384 (11.5%) | 10,686 (11.4%) | 38,536 (7.3%) | 39,191 (7.2%) | 30,424 (5.5%) | 30,858 (5.5%) |
| Urban | 3,296 (8.0%) | 3,386 (8.0%) | 7,165  (4.5%) | 7,269  (4.5%) | 4,906  (3.5%) | 4,976  (3.5%) |
| Rural | 7,088 (14.5%) | 7,300 (14.4%) | 31,371 (8.4%) | 31,922 (8.4%) | 25,518 (6.2%) | 25,882 (6.2%) |
| **Mildly Thin** |  |  |  |  |  |  |
| All | 13,683 (15.1%) | 14,174 (15.2%) | 60,255 (11.3%) | 61,285 (11.3%) | 47,791 (8.7%) | 48,524 (8.7%) |
| Urban | 4,327 (10.5%) | 4,456 (10.5%) | 11,300 (7.1%) | 11,474 (7.1%) | 7,554  (5.4%) | 7,680  (5.5%) |
| Rural | 9,356 (19.1%) | 9,718 (19.1%) | 48,955 (13.1%) | 49,811 (13.1%) | 40,237 (9.8%) | 40,844 (9.8%) |
| **Normal** |  |  |  |  |  |  |
| All | 49,755 (55.1%) | 51,677 (55.3%) | 316,746 (59.6%) | 323,163 (59.7%) | 331,220 (60.1%) | 336,617 (60.2%) |
| Urban | 22,273 (53.9%) | 23,060 (54.1%) | 87,600 (55.2%) | 89,273 (55.3%) | 75,737 (54.6%) | 76,856 (54.7%) |
| Rural | 27,482 (56.1%) | 28,617 (56.3%) | 229,146 (61.5%) | 233,890 (61.6%) | 255,483 (62.0%) | 259,761 (62.1%) |
| **Overweight** |  |  |  |  |  |  |
| All | 12,624 (14.0%) | 12,934 (13.8%) | 88,673 (16.7%) | 89,827 (16.6%) | 106,652 (19.4%) | 107,829 (19.3%) |
| Urban | 8,433 (20.4%) | 8,631 (20.3%) | 37,978 (23.9%) | 38,470 (23.8%) | 35,294 (25.4%) | 35,634 (25.4%) |
| Rural | 4,191 (8.6%) | 4,303 (8.5%) | 50,695 (13.6%) | 51,357 (13.5%) | 71,358 (17.3%) | 72,195 (17.2%) |
| **Obese** |  |  |  |  |  |  |
| All | 3,887 (4.3%) | 3,965 (4.2%) | 27,223 (5.1%) | 27,514 (5.1%) | 34,940 (6.3%) | 35,270 (6.3%) |
| Urban | 3,000 (7.3%) | 3,053 (7.2%) | 14,693 (9.3%) | 14,836 (9.2%) | 15,222 (11.0%) | 15,361 (10.9%) |
| Rural | 887  (1.8%) | 912  (1.8%) | 12,530 (3.4%) | 12,678 (3.3%) | 19,718 (4.8%) | 19,909 (4.8%) |

**Note: ***IR refers to the Individual Recode dataset. **PR refers to the Household Member Recode dataset.

**Table S2: State-level Spearman’s Rank Correlation (p value) of BMI outcomes across surveys, Women 1999-2021 (A) and Men 2006-2021 (B)**

A. Women 1999-2021

| **Year** | **1999**  **(n=71385)** | **2006**  **(n=90333)** | **2016**  **(n=531433)** | **2021**  **(n=551027)** |
| --- | --- | --- | --- | --- |
| **Severely/Moderately Thin** | | | | |
| **1999** | 1 |  |  |  |
| **2006** | 0.85* | 1 |  |  |
| **2016** | 0.80* | 0.92* | 1 |  |
| **2021** | 0.82* | 0.91* | 0.96* | 1 |
| **Mildly Thin** | | | | |
| **1999** | 1 |  |  |  |
| **2006** | 0.91* | 1 |  |  |
| **2016** | 0.88* | 0.95* | 1 |  |
| **2021** | 0.84* | 0.90* | 0.94* | 1 |
| **Normal** | | | | |
| **1999** | 1 |  |  |  |
| **2006** | 0.81* | 1 |  |  |
| **2016** | 0.66* | 0.64* | 1 |  |
| **2021** | 0.40* | 0.51 (p=0.004) | 0.74* | 1 |
| **Overweight** | | | | |
| **1999** | 1 |  |  |  |
| **2006** | 0.94* | 1 |  |  |
| **2016** | 0.88* | 0.92* | 1 |  |
| **2021** | 0.83* | 0.87* | 0.89* | 1 |
| **Obese** | | | | |
| **1999** | 1 |  |  |  |
| **2006** | 0.88* | 1 |  |  |
| **2016** | 0.77* | 0.90* | 1 |  |
| **2021** | 0.77* | 0.90* | 0.90* | 1 |

**Note:** An asterisk (*) indicates a p-value of less than 0.001.

B. Men 2006 - 2021

| **Year** | **2006**  **(n=58935)** | **2016**  **(n=92574)** | **2021**  **(n=82198)** |
| --- | --- | --- | --- |
| **Severely/Moderately Thin** | | | |
| **2006** | 1 |  |  |
| **2016** | 0.80* | 1 |  |
| **2021** | 0.76* | 0.86* | 1 |
| **Mildly Thin** | | | |
| **2006** | 1 |  |  |
| **2016** | 0.86* | 1 |  |
| **2021** | 0.82* | 0.86* | 1 |
| **Normal** | | | |
| **2006** | 1 |  |  |
| **2016** | 0.48 (p=0.006) | 1 |  |
| **2021** | -0.04 (p=0.84) | 0.50 (p=0.004) | 1 |
| **Overweight** | | | |
| **2006** | 1 |  |  |
| **2016** | 0.91* | 1 |  |
| **2021** | 0.76* | 0.84* | 1 |
| **Obese** | | | |
| **2006** | 1 |  |  |
| **2016** | 0.74* | 1 |  |
| **2021** | 0.78* | 0.78* | 1 |

**Note:** An asterisk (*) indicates a p-value of less than 0.001.

**Figure S1: Comparative Distribution of Body Mass Index (BMI) Across States and Union Territories by region: Earliest and Latest Survey Period (2021)**

**A.** Women

**Note:** The upper bar represents the BMI distribution for the earliest period, while the lower bar depicts the BMI distribution for the latest period (2021). The cutoff points of BMI categories are: Severely/Moderately Thin (<17.0), Mildly Thin (16.0-18.4), Normal (18.5-24.9), Overweight (25.0-29.9), and Obese (≥30.0). The earliest survey period for Ladakh is 2006. The earliest survey period for Andaman & Nicobar, Chandigarh, Dagar and Nagar Havali and Diu, Lakshadweep, and Puducherry is 2016. The earliest survey period for all other states is 1999.

**B.** Women, Rural-India

**Note:** The upper bar represents the BMI distribution for the earliest period, while the lower bar depicts the BMI distribution for the latest period (2021). The cutoff points of BMI categories are: Severely/Moderately Thin (<17.0), Mildly Thin (16.0-18.4), Normal (18.5-24.9), Overweight (25.0-29.9), and Obese (≥30.0). The earliest survey period for Ladakh is 2006. The earliest survey period for Andaman & Nicobar, Chandigarh, Dagar and Nagar Havali and Diu, Lakshadweep, and Puducherry is 2016. The earliest survey period for all other states is 1999.

**C.** Men, Urban-India

**Note:** The upper bar represents the BMI distribution for the earliest period, while the lower bar depicts the BMI distribution for the latest period (2021). The cutoff points of BMI categories are: Severely/Moderately Thin (<17.0), Mildly Thin (16.0-18.4), Normal (18.5-24.9), Overweight (25.0-29.9), and Obese (≥30.0). The earliest survey period for Andaman & Nicobar, Chandigarh, Dagar and Nagar Havali and Diu, Lakshadweep, and Puducherry is 2016. The earliest survey period for all other states is 2006.

**D.** Men, Rural-India

**Note:** The upper bar represents the BMI distribution for the earliest period, while the lower bar depicts the BMI distribution for the latest period (2021). The cutoff points of BMI categories are: Severely/Moderately Thin (<17.0), Mildly Thin (16.0-18.4), Normal (18.5-24.9), Overweight (25.0-29.9), and Obese (≥30.0). The earliest survey period for Andaman & Nicobar, Chandigarh, Dagar and Nagar Havali and Diu, Lakshadweep, and Puducherry is 2016. The earliest survey period for all other states is 2006.

**Figure S2: Summary distribution of state/Union Territory-level Body Mass Index (BMI) category by region of India**

**A.** Women, 1999-2021, Urban-India

**B.** Women, 1999-2021, Rural-India

**C.** Men, 2006-2021, Urban-India

**D.** Men, 2006-2021, Rural-India

**Figure S3: Weighted Prevalence of Body Mass Index (BMI) categories of India and State/Union Territories, Women (A) and Men (B)**

A. Women, 1999-2021

|  | **Severely/Moderately Thin** | | | | | | | | **Mildly Thin** | | | | | | | | **Normal** | | | | | | | | **Overweight** | | | | | | | | **Obese** | | | | | | | |
| --- | --- | --- | --- | --- | --- | --- | --- | --- | --- | --- | --- | --- | --- | --- | --- | --- | --- | --- | --- | --- | --- | --- | --- | --- | --- | --- | --- | --- | --- | --- | --- | --- | --- | --- | --- | --- | --- | --- | --- | --- |
|  | <5 | 5-  9.9 | | 10-  14.9 | | 15-  19.9 | | ≥20 | <5 | 5-  9.9 | | 10-  14.9 | | 15-  19.9 | | ≥20 | <50 | 50-  54.9 | | 55-  59.9 | | 60-  69.9 | | ≥70 | <5 | 5-  9.9 | | 10-  14.9 | | 15-  19.9 | | ≥20 | <5 | 5-  9.9 | | 10-  14.9 | | 15-  19.9 | | ≥20 |
|  |  | |  | |  | |  | |  | |  | |  | |  | |  | |  | |  | |  | |  | |  | |  | |  | |  | |  | |  | |  | |
|  | **1999** | | **2006** | | **2016** | | **2021** | | **1999** | | **2006** | | **2016** | | **2021** | | **1999** | | **2006** | | **2016** | | **2021** | | **1999** | | **2006** | | **2016** | | **2021** | | **1999** | | **2006** | | **2016** | | **2021** | |
| **All-India** | 15.9 | | 14.6 | | 7.6 | | 5.7 | | 19.9 | | 18.2 | | 11.2 | | 8.7 | | 52.7 | | 52.0 | | 57.0 | | 57.8 | | 9.0 | | 11.7 | | 18.1 | | 20.4 | | 2.4 | | 3.5 | | 6.1 | | 7.5 | |
| Andhra Pradesh | 17.1 | | 14.5 | | 5.7 | | 4.8 | | 19.3 | | 16.7 | | 8.8 | | 6.7 | | 51.0 | | 50.6 | | 48.7 | | 48.7 | | 10.0 | | 13.2 | | 25.2 | | 26.5 | | 2.6 | | 4.9 | | 11.5 | | 13.3 | |
| Arunachal Pradesh | 3.2 | | 4.8 | | 1.9 | | 1.2 | | 7.7 | | 10.1 | | 4.9 | | 2.9 | | 83.8 | | 74.1 | | 71.8 | | 68.5 | | 4.7 | | 9.5 | | 18.0 | | 22.9 | | 0.6 | | 1.4 | | 3.4 | | 4.4 | |
| Assam | 9.7 | | 15.1 | | 9.2 | | 5.7 | | 17.3 | | 20.2 | | 13.9 | | 9.1 | | 68.6 | | 55.4 | | 61.8 | | 67.9 | | 3.6 | | 8.1 | | 12.8 | | 14.4 | | 0.7 | | 1.1 | | 2.4 | | 2.9 | |
| Bihar | 16.4 | | 18.8 | | 9.9 | | 8.0 | | 23.1 | | 23.9 | | 16.2 | | 12.1 | | 56.3 | | 51.5 | | 59.3 | | 59.9 | | 3.6 | | 4.8 | | 11.7 | | 15.9 | | 0.6 | | 0.8 | | 2.8 | | 4.1 | |
| Chhattisgarh | 20.3 | | 18.1 | | 8.6 | | 7.2 | | 28.4 | | 23.1 | | 14.9 | | 12.2 | | 46.7 | | 52.0 | | 62.4 | | 64.2 | | 3.5 | | 5.3 | | 11.3 | | 12.6 | | 1.1 | | 1.6 | | 2.8 | | 3.8 | |
| Goa | 12.8 | | 12.6 | | 4.9 | | 4.3 | | 14.3 | | 11.8 | | 6.1 | | 5.2 | | 51.2 | | 52.6 | | 51.2 | | 50.6 | | 17.1 | | 17.7 | | 27.0 | | 29.2 | | 4.5 | | 5.4 | | 10.9 | | 10.7 | |
| Gujarat | 18.7 | | 16.3 | | 10.8 | | 9.3 | | 18.0 | | 16.1 | | 12.0 | | 10.8 | | 46.7 | | 47.7 | | 49.8 | | 54.0 | | 12.0 | | 14.4 | | 19.3 | | 18.1 | | 4.7 | | 5.5 | | 8.2 | | 7.9 | |
| Haryana | 10.4 | | 12.4 | | 4.6 | | 4.0 | | 15.7 | | 15.4 | | 7.2 | | 6.2 | | 56.6 | | 51.1 | | 63.9 | | 51.7 | | 13.3 | | 15.9 | | 18.6 | | 26.6 | | 4.1 | | 5.3 | | 5.6 | | 11.5 | |
| Himachal Pradesh | 13.4 | | 10.5 | | 4.3 | | 4.3 | | 16.2 | | 14.5 | | 7.8 | | 5.5 | | 57.0 | | 59.0 | | 55.4 | | 55.7 | | 11.0 | | 13.5 | | 24.6 | | 26.0 | | 2.4 | | 2.5 | | 8.0 | | 8.5 | |
| Jharkhand | 16.9 | | 17.7 | | 11.5 | | 8.0 | | 24.5 | | 24.0 | | 17.1 | | 14.3 | | 55.2 | | 51.8 | | 59.1 | | 63.7 | | 3.0 | | 5.4 | | 9.8 | | 11.0 | | 0.4 | | 1.2 | | 2.5 | | 3.0 | |
| Karnataka | 18.3 | | 14.8 | | 7.4 | | 5.4 | | 20.0 | | 17.0 | | 9.6 | | 7.5 | | 46.9 | | 50.2 | | 56.9 | | 53.2 | | 11.6 | | 13.5 | | 18.7 | | 24.2 | | 3.2 | | 4.5 | | 7.5 | | 9.6 | |
| Kerala | 6.8 | | 7.0 | | 2.2 | | 2.5 | | 12.2 | | 7.8 | | 4.1 | | 3.7 | | 59.8 | | 53.3 | | 57.4 | | 50.7 | | 17.2 | | 26.3 | | 29.1 | | 32.0 | | 4.0 | | 5.6 | | 7.2 | | 11.1 | |
| Madhya Pradesh | 14.2 | | 17.7 | | 9.5 | | 6.7 | | 20.9 | | 22.6 | | 14.9 | | 11.7 | | 57.4 | | 50.8 | | 59.4 | | 62.0 | | 6.1 | | 7.3 | | 12.5 | | 15.1 | | 1.4 | | 1.6 | | 3.7 | | 4.5 | |
| Maharashtra | 19.9 | | 15.1 | | 8.5 | | 7.1 | | 19.2 | | 17.5 | | 11.0 | | 9.3 | | 48.1 | | 50.2 | | 53.8 | | 57.5 | | 9.6 | | 12.9 | | 19.7 | | 19.1 | | 3.2 | | 4.3 | | 7.0 | | 7.0 | |
| Manipur | 6.2 | | 2.8 | | 1.5 | | 1.2 | | 13.3 | | 11.0 | | 5.3 | | 3.8 | | 69.7 | | 70.8 | | 63.7 | | 56.8 | | 9.7 | | 13.2 | | 23.5 | | 29.8 | | 1.2 | | 2.2 | | 6.1 | | 8.4 | |
| Meghalaya | 10.8 | | 4.7 | | 3.0 | | 2.3 | | 14.5 | | 9.5 | | 8.4 | | 6.4 | | 68.8 | | 79.2 | | 74.2 | | 77.6 | | 4.7 | | 5.6 | | 12.4 | | 12.1 | | 1.2 | | 1.0 | | 2.1 | | 1.6 | |
| Mizoram | 6.1 | | 4.2 | | 1.4 | | 1.0 | | 16.9 | | 10.1 | | 6.0 | | 3.4 | | 71.6 | | 73.7 | | 68.6 | | 69.0 | | 4.9 | | 10.5 | | 19.7 | | 21.5 | | 0.5 | | 1.5 | | 4.3 | | 5.2 | |
| Nagaland | 5.8 | | 3.6 | | 2.2 | | 2.2 | | 12.0 | | 12.0 | | 7.8 | | 4.8 | | 73.1 | | 76.7 | | 71.4 | | 76.5 | | 8.3 | | 6.9 | | 15.5 | | 14.5 | | 0.8 | | 0.8 | | 3.1 | | 2.0 | |
| Odisha | 21.1 | | 18.4 | | 10.0 | | 7.2 | | 27.1 | | 22.2 | | 14.1 | | 11.2 | | 47.2 | | 51.8 | | 57.1 | | 55.9 | | 4.0 | | 6.2 | | 14.8 | | 20.0 | | 0.6 | | 1.4 | | 4.0 | | 5.7 | |
| Punjab | 7.0 | | 5.4 | | 3.0 | | 3.5 | | 9.7 | | 9.1 | | 4.8 | | 5.1 | | 52.2 | | 50.2 | | 56.6 | | 45.4 | | 21.8 | | 24.2 | | 25.1 | | 30.0 | | 9.3 | | 11.0 | | 10.6 | | 16.1 | |
| Rajasthan | 14.2 | | 13.3 | | 9.0 | | 5.3 | | 22.6 | | 20.2 | | 13.4 | | 9.2 | | 55.6 | | 55.8 | | 60.6 | | 69.9 | | 5.8 | | 8.5 | | 13.0 | | 12.3 | | 1.8 | | 2.2 | | 4.0 | | 3.3 | |
| Sikkim | 2.7 | | 2.6 | | 1.1 | | 1.6 | | 8.4 | | 6.6 | | 3.5 | | 3.0 | | 72.9 | | 72.6 | | 64.7 | | 57.2 | | 13.5 | | 14.5 | | 24.3 | | 30.5 | | 2.6 | | 3.7 | | 6.4 | | 7.6 | |
| Tamil Nadu | 13.6 | | 11.5 | | 4.6 | | 3.7 | | 15.2 | | 13.8 | | 6.3 | | 5.5 | | 56.1 | | 51.2 | | 54.4 | | 46.3 | | 12.3 | | 17.8 | | 25.4 | | 29.0 | | 2.8 | | 5.8 | | 9.4 | | 15.5 | |
| Telangana | 16.9 | | 14.5 | | 8.6 | | 6.8 | | 19.9 | | 16.7 | | 10.5 | | 8.7 | | 48.6 | | 50.6 | | 49.1 | | 51.4 | | 12.1 | | 13.2 | | 22.1 | | 23.2 | | 2.4 | | 4.9 | | 9.8 | | 9.9 | |
| Tripura | 13.5 | | 15.8 | | 6.3 | | 4.8 | | 21.8 | | 19.7 | | 11.1 | | 8.7 | | 56.0 | | 56.3 | | 64.9 | | 62.8 | | 6.8 | | 7.3 | | 15.1 | | 19.4 | | 1.9 | | 0.9 | | 2.6 | | 4.2 | |
| Uttar Pradesh | 14.9 | | 14.4 | | 7.8 | | 5.0 | | 21.9 | | 19.5 | | 12.5 | | 8.9 | | 55.1 | | 54.3 | | 59.0 | | 59.9 | | 6.5 | | 9.5 | | 15.8 | | 19.7 | | 1.6 | | 2.3 | | 4.9 | | 6.5 | |
| Uttarakhand | 9.2 | | 9.7 | | 4.8 | | 3.2 | | 21.0 | | 16.7 | | 10.2 | | 7.1 | | 58.0 | | 58.0 | | 60.9 | | 55.2 | | 8.1 | | 12.3 | | 18.1 | | 25.7 | | 3.8 | | 3.2 | | 5.9 | | 8.8 | |
| West Bengal | 21.6 | | 16.8 | | 7.3 | | 4.7 | | 23.0 | | 19.9 | | 10.6 | | 7.1 | | 46.7 | | 49.9 | | 59.5 | | 62.6 | | 7.4 | | 11.0 | | 18.2 | | 20.5 | | 1.3 | | 2.4 | | 4.3 | | 5.2 | |
| NCT Delhi(UT) | 3.7 | | 3.9 | | 3.7 | | 2.4 | | 8.1 | | 7.3 | | 5.9 | | 3.6 | | 53.7 | | 57.9 | | 51.6 | | 46.9 | | 25.1 | | 21.4 | | 26.9 | | 30.7 | | 9.5 | | 9.5 | | 11.9 | | 16.4 | |
| Jammu&Kashmir (UT) | 10.0 | | 8.0 | | 2.8 | | 0.9 | | 16.4 | | 13.7 | | 6.3 | | 2.0 | | 59.5 | | 58.3 | | 57.1 | | 63.6 | | 11.0 | | 15.8 | | 24.8 | | 28.0 | | 3.1 | | 4.2 | | 8.9 | | 5.5 | |
| Ladakh(UT) |  | | 8.0 | | 1.3 | | 0.5 | |  | | 13.7 | | 6.5 | | 1.4 | |  | | 58.3 | | 73.7 | | 66.1 | |  | | 15.8 | | 15.9 | | 28.4 | |  | | 4.2 | | 2.5 | | 3.6 | |
| Andaman&Nicobar (UT) |  | |  | | 5.4 | | 2.6 | |  | |  | | 5.5 | | 4.0 | |  | |  | | 53.7 | | 51.3 | |  | |  | | 26.6 | | 28.0 | |  | |  | | 8.8 | | 14.0 | |
| Chandigarh(UT) |  | |  | | 4.6 | | 3.7 | |  | |  | | 4.5 | | 4.4 | |  | |  | | 43.4 | | 42.2 | |  | |  | | 30.2 | | 30.6 | |  | |  | | 17.3 | | 19.0 | |
| Dadra and Nagar Haveli and Diu(UT) |  | |  | | 8.7 | | 9.8 | |  | |  | | 11.0 | | 11.3 | |  | |  | | 53.9 | | 49.4 | |  | |  | | 19.4 | | 21.4 | |  | |  | | 7.0 | | 8.2 | |
| Lakshadweep(UT) |  | |  | | 5.8 | | 2.9 | |  | |  | | 5.4 | | 2.3 | |  | |  | | 43.7 | | 56.8 | |  | |  | | 29.1 | | 30.1 | |  | |  | | 15.9 | | 7.9 | |
| Puducherry(UT) |  | |  | | 2.3 | | 2.2 | |  | |  | | 4.1 | | 3.7 | |  | |  | | 52.4 | | 42.5 | |  | |  | | 29.6 | | 31.4 | |  | |  | | 11.6 | | 20.2 | |

|  | **Severely/Moderately Thin** | | | | | | | | **Mildly Thin** | | | | | | | | **Normal** | | | | | | | | **Overweight** | | | | | | | | **Obese** | | | | | | | |
| --- | --- | --- | --- | --- | --- | --- | --- | --- | --- | --- | --- | --- | --- | --- | --- | --- | --- | --- | --- | --- | --- | --- | --- | --- | --- | --- | --- | --- | --- | --- | --- | --- | --- | --- | --- | --- | --- | --- | --- | --- |
|  | <5 | 5-  9.9 | | 10-  14.9 | | 15-  19.9 | | ≥20 | <5 | 5-  9.9 | | 10-  14.9 | | 15-  19.9 | | ≥20 | <50 | 50-  54.9 | | 55-  59.9 | | 60-  69.9 | | ≥70 | <5 | 5-  9.9 | | 10-  14.9 | | 15-  19.9 | | ≥20 | <5 | 5-  9.9 | | 10-  14.9 | | 15-  19.9 | | ≥20 |
|  |  | |  | |  | |  | |  | |  | |  | |  | |  | |  | |  | |  | |  | |  | |  | |  | |  | |  | |  | |  | |
|  | **1999** | | **2006** | | **2016** | | **2021** | | **1999** | | **2006** | | **2016** | | **2021** | | **1999** | | **2006** | | **2016** | | **2021** | | **1999** | | **2006** | | **2016** | | **2021** | | **1999** | | **2006** | | **2016** | | **2021** | |
| **Urban-India** | 9.7 | | 9.3 | | 4.6 | | 3.7 | | 12.3 | | 11.4 | | 6.8 | | 5.5 | | 53.4 | | 51.6 | | 52.9 | | 53.4 | | 18.4 | | 20.3 | | 25.2 | | 25.7 | | 6.2 | | 7.3 | | 10.5 | | 11.8 | |
| Andhra Pradesh | 10.3 | | 7.9 | | 3.2 | | 3.3 | | 10.9 | | 10.0 | | 5.1 | | 5.0 | | 57.5 | | 51.0 | | 41.4 | | 43.0 | | 17.1 | | 21.7 | | 31.7 | | 30.3 | | 4.2 | | 9.4 | | 18.5 | | 18.3 | |
| Arunachal Pradesh | 2.5 | | 6.6 | | 1.9 | | 1.6 | | 9.8 | | 10.3 | | 3.9 | | 2.3 | | 80.3 | | 68.3 | | 64.2 | | 63.2 | | 7.4 | | 12.3 | | 24.4 | | 26.4 | | 0.0 | | 2.6 | | 5.6 | | 6.5 | |
| Assam | 5.6 | | 9.7 | | 5.9 | | 4.2 | | 12.7 | | 13.2 | | 8.2 | | 7.1 | | 68.2 | | 54.6 | | 57.1 | | 62.4 | | 11.4 | | 19.0 | | 22.3 | | 20.4 | | 2.2 | | 3.6 | | 6.6 | | 5.9 | |
| Bihar | 13.7 | | 10.8 | | 6.4 | | 5.0 | | 17.9 | | 14.7 | | 9.3 | | 7.5 | | 56.7 | | 58.9 | | 55.4 | | 56.9 | | 9.2 | | 11.8 | | 21.5 | | 23.1 | | 2.5 | | 3.7 | | 7.3 | | 7.5 | |
| Chhattisgarh | 17.7 | | 11.1 | | 5.0 | | 4.1 | | 15.6 | | 13.3 | | 8.7 | | 7.2 | | 53.1 | | 55.1 | | 58.1 | | 62.3 | | 10.2 | | 15.1 | | 21.1 | | 18.9 | | 3.4 | | 5.4 | | 7.1 | | 7.6 | |
| Goa | 10.5 | | 9.9 | | 3.0 | | 3.8 | | 11.9 | | 10.2 | | 3.9 | | 5.5 | | 50.6 | | 52.1 | | 51.7 | | 48.9 | | 20.0 | | 20.6 | | 28.1 | | 30.1 | | 7.0 | | 7.2 | | 13.2 | | 11.8 | |
| Gujarat | 11.1 | | 9.4 | | 5.8 | | 5.2 | | 11.3 | | 10.6 | | 8.1 | | 7.4 | | 49.8 | | 49.6 | | 47.0 | | 53.3 | | 19.4 | | 21.5 | | 26.2 | | 22.9 | | 8.4 | | 9.0 | | 12.9 | | 11.2 | |
| Haryana | 5.6 | | 7.1 | | 3.0 | | 3.0 | | 7.6 | | 9.7 | | 5.5 | | 4.4 | | 51.4 | | 49.0 | | 63.8 | | 50.4 | | 25.7 | | 24.2 | | 20.6 | | 28.1 | | 9.7 | | 9.9 | | 7.1 | | 14.2 | |
| Himachal Pradesh | 5.2 | | 5.0 | | 2.7 | | 3.1 | | 12.4 | | 7.9 | | 4.8 | | 2.9 | | 50.3 | | 53.7 | | 49.6 | | 51.5 | | 23.5 | | 25.8 | | 31.3 | | 30.7 | | 8.6 | | 7.7 | | 11.7 | | 11.8 | |
| Jharkhand | 13.1 | | 11.6 | | 7.2 | | 4.3 | | 16.8 | | 14.4 | | 10.2 | | 7.8 | | 61.1 | | 54.7 | | 56.8 | | 62.6 | | 7.7 | | 15.3 | | 19.2 | | 19.0 | | 1.4 | | 4.0 | | 6.7 | | 6.3 | |
| Karnataka | 10.0 | | 10.4 | | 5.5 | | 3.7 | | 12.5 | | 10.4 | | 7.0 | | 5.1 | | 49.0 | | 48.5 | | 52.1 | | 49.9 | | 21.1 | | 21.6 | | 23.7 | | 28.0 | | 7.3 | | 9.2 | | 11.7 | | 13.3 | |
| Kerala | 5.9 | | 6.0 | | 2.0 | | 2.4 | | 8.2 | | 6.7 | | 3.3 | | 3.1 | | 60.4 | | 51.0 | | 57.5 | | 48.6 | | 20.0 | | 29.9 | | 29.5 | | 32.9 | | 5.4 | | 6.4 | | 7.7 | | 13.0 | |
| Madhya Pradesh | 11.2 | | 13.2 | | 6.2 | | 4.3 | | 15.4 | | 15.8 | | 9.6 | | 8.1 | | 55.5 | | 48.8 | | 56.5 | | 57.6 | | 14.3 | | 17.2 | | 20.1 | | 22.1 | | 3.7 | | 5.0 | | 7.5 | | 7.9 | |
| Maharashtra | 12.8 | | 10.1 | | 5.3 | | 4.7 | | 12.5 | | 11.5 | | 7.5 | | 6.6 | | 50.6 | | 52.2 | | 50.5 | | 56.3 | | 17.8 | | 18.8 | | 26.0 | | 22.3 | | 6.3 | | 7.4 | | 10.6 | | 10.1 | |
| Manipur | 6.6 | | 2.8 | | 1.3 | | 1.0 | | 12.9 | | 8.6 | | 4.8 | | 2.9 | | 64.6 | | 66.1 | | 58.3 | | 53.3 | | 14.3 | | 18.7 | | 27.2 | | 32.3 | | 1.5 | | 3.9 | | 8.4 | | 10.4 | |
| Meghalaya | 7.0 | | 4.8 | | 2.5 | | 2.5 | | 13.1 | | 11.0 | | 7.8 | | 6.4 | | 66.1 | | 74.4 | | 67.9 | | 69.9 | | 8.5 | | 9.0 | | 17.2 | | 17.8 | | 5.4 | | 0.8 | | 4.6 | | 3.4 | |
| Mizoram | 4.0 | | 2.9 | | 0.9 | | 0.8 | | 12.4 | | 8.1 | | 5.3 | | 2.8 | | 76.3 | | 72.0 | | 63.2 | | 63.8 | | 6.8 | | 14.7 | | 25.1 | | 25.6 | | 0.6 | | 2.4 | | 5.5 | | 7.1 | |
| Nagaland | 7.6 | | 4.0 | | 2.4 | | 2.4 | | 5.1 | | 9.9 | | 7.4 | | 4.5 | | 71.7 | | 71.1 | | 66.3 | | 73.6 | | 14.2 | | 13.0 | | 19.3 | | 16.7 | | 1.4 | | 2.0 | | 4.5 | | 2.8 | |
| Odisha | 15.4 | | 10.9 | | 4.6 | | 3.8 | | 16.8 | | 14.3 | | 7.6 | | 5.5 | | 54.2 | | 54.1 | | 51.6 | | 46.8 | | 11.1 | | 16.7 | | 25.7 | | 31.0 | | 2.5 | | 4.0 | | 10.5 | | 12.8 | |
| Punjab | 3.3 | | 4.8 | | 2.0 | | 3.2 | | 5.7 | | 8.1 | | 3.8 | | 4.2 | | 46.2 | | 45.7 | | 58.2 | | 42.8 | | 28.4 | | 27.2 | | 25.3 | | 31.9 | | 16.4 | | 14.3 | | 10.8 | | 17.9 | |
| Rajasthan | 11.0 | | 10.9 | | 5.6 | | 3.6 | | 17.2 | | 15.1 | | 8.2 | | 5.6 | | 52.7 | | 52.7 | | 58.6 | | 66.8 | | 13.9 | | 16.3 | | 19.3 | | 17.5 | | 5.1 | | 4.9 | | 8.2 | | 6.4 | |
| Sikkim | 4.1 | | 1.9 | | 1.1 | | 1.3 | | 8.2 | | 4.0 | | 3.5 | | 3.6 | | 60.8 | | 69.6 | | 57.1 | | 51.2 | | 24.7 | | 18.8 | | 28.5 | | 34.3 | | 2.1 | | 5.7 | | 9.7 | | 9.6 | |
| Tamil Nadu | 6.4 | | 8.4 | | 3.1 | | 2.8 | | 10.6 | | 10.7 | | 4.4 | | 3.8 | | 57.7 | | 48.0 | | 51.9 | | 42.8 | | 20.1 | | 23.6 | | 28.5 | | 30.8 | | 5.3 | | 9.3 | | 12.1 | | 19.8 | |
| Telangana | 6.5 | | 7.9 | | 5.5 | | 4.3 | | 8.4 | | 10.0 | | 6.5 | | 5.6 | | 52.0 | | 51.0 | | 43.6 | | 44.4 | | 26.4 | | 21.7 | | 28.4 | | 29.1 | | 6.7 | | 9.4 | | 15.9 | | 16.6 | |
| Tripura | 8.4 | | 10.1 | | 5.3 | | 3.5 | | 18.5 | | 14.8 | | 9.4 | | 8.1 | | 53.5 | | 57.5 | | 60.0 | | 57.2 | | 17.8 | | 14.2 | | 20.8 | | 23.5 | | 1.8 | | 3.4 | | 4.5 | | 7.7 | |
| Uttar Pradesh | 9.3 | | 11.0 | | 4.9 | | 3.2 | | 14.6 | | 12.0 | | 7.6 | | 5.6 | | 56.0 | | 53.2 | | 54.8 | | 55.0 | | 14.8 | | 18.0 | | 23.0 | | 25.4 | | 5.4 | | 5.7 | | 9.7 | | 10.7 | |
| Uttarakhand | 4.5 | | 4.5 | | 4.4 | | 2.6 | | 12.0 | | 9.3 | | 8.2 | | 5.4 | | 54.9 | | 58.1 | | 54.9 | | 47.5 | | 18.9 | | 22.3 | | 23.8 | | 30.8 | | 9.6 | | 5.8 | | 8.7 | | 13.7 | |
| West Bengal | 11.0 | | 8.6 | | 4.0 | | 3.1 | | 13.5 | | 11.3 | | 6.6 | | 3.9 | | 51.2 | | 51.7 | | 55.4 | | 62.2 | | 19.1 | | 22.1 | | 26.2 | | 23.4 | | 5.0 | | 6.3 | | 7.9 | | 7.4 | |
| NCT Delhi(UT) | 3.7 | | 3.6 | | 3.7 | | 2.4 | | 8.0 | | 7.2 | | 5.9 | | 3.6 | | 53.1 | | 57.5 | | 51.5 | | 46.9 | | 25.3 | | 21.9 | | 26.9 | | 30.7 | | 9.9 | | 9.8 | | 11.9 | | 16.4 | |
| Jammu&Kashmir (UT) | 4.4 | | 4.1 | | 1.4 | | 0.7 | | 8.4 | | 7.8 | | 3.9 | | 1.3 | | 55.2 | | 52.8 | | 49.4 | | 60.7 | | 23.9 | | 26.8 | | 32.9 | | 29.8 | | 8.1 | | 8.5 | | 12.4 | | 7.5 | |
| Ladakh(UT) |  | | 4.1 | | 1.0 | | 0.5 | |  | | 7.8 | | 4.1 | | 1.2 | |  | | 52.8 | | 71.0 | | 66.3 | |  | | 26.8 | | 20.0 | | 27.7 | |  | | 8.5 | | 3.9 | | 4.4 | |
| Andaman&Nicobar (UT) |  | |  | | 4.5 | | 3.9 | |  | |  | | 4.4 | | 4.5 | |  | |  | | 49.1 | | 45.7 | |  | |  | | 30.6 | | 29.6 | |  | |  | | 11.4 | | 16.3 | |
| Chandigarh(UT) |  | |  | | 4.6 | | 3.8 | |  | |  | | 3.8 | | 4.4 | |  | |  | | 43.0 | | 42.4 | |  | |  | | 31.1 | | 30.5 | |  | |  | | 17.5 | | 19.0 | |
| Dadra and Nagar Haveli and Diu(UT) |  | |  | | 4.7 | | 8.4 | |  | |  | | 6.2 | | 8.1 | |  | |  | | 51.7 | | 45.7 | |  | |  | | 26.7 | | 25.8 | |  | |  | | 10.7 | | 12.0 | |
| Lakshadweep(UT) |  | |  | | 5.3 | | 2.5 | |  | |  | | 4.7 | | 1.5 | |  | |  | | 43.4 | | 57.3 | |  | |  | | 30.5 | | 30.6 | |  | |  | | 16.1 | | 8.2 | |
| Puducherry(UT) |  | |  | | 2.0 | | 1.5 | |  | |  | | 3.7 | | 3.6 | |  | |  | | 51.7 | | 41.7 | |  | |  | | 30.1 | | 30.8 | |  | |  | | 12.5 | | 22.4 | |

|  | **Severely/Moderately Thin** | | | | | | | | **Mildly Thin** | | | | | | | | **Normal** | | | | | | | | **Overweight** | | | | | | | | **Obese** | | | | | | | |
| --- | --- | --- | --- | --- | --- | --- | --- | --- | --- | --- | --- | --- | --- | --- | --- | --- | --- | --- | --- | --- | --- | --- | --- | --- | --- | --- | --- | --- | --- | --- | --- | --- | --- | --- | --- | --- | --- | --- | --- | --- |
|  | <5 | 5-  9.9 | | 10-  14.9 | | 15-  19.9 | | ≥20 | <5 | 5-  9.9 | | 10-  14.9 | | 15-  19.9 | | ≥20 | <50 | 50-  54.9 | | 55-  59.9 | | 60-  69.9 | | ≥70 | <5 | 5-  9.9 | | 10-  14.9 | | 15-  19.9 | | ≥20 | <5 | 5-  9.9 | | 10-  14.9 | | 15-  19.9 | | ≥20 |
|  |  | |  | |  | |  | |  | |  | |  | |  | |  | |  | |  | |  | |  | |  | |  | |  | |  | |  | |  | |  | |
|  | **1999** | | **2006** | | **2016** | | **2021** | | **1999** | | **2006** | | **2016** | | **2021** | | **1999** | | **2006** | | **2016** | | **2021** | | **1999** | | **2006** | | **2016** | | **2021** | | **1999** | | **2006** | | **2016** | | **2021** | |
| **Rural-India** | 18.3 | | 17.2 | | 9.3 | | 6.7 | | 22.8 | | 21.6 | | 13.6 | | 10.3 | | 52.4 | | 52.2 | | 59.3 | | 59.9 | | 5.4 | | 7.4 | | 14.2 | | 17.8 | | 1.0 | | 1.6 | | 3.6 | | 5.3 | |
| Andhra Pradesh | 19.1 | | 17.7 | | 6.9 | | 5.5 | | 21.7 | | 20.1 | | 10.4 | | 7.5 | | 49.1 | | 50.4 | | 52.0 | | 51.2 | | 7.9 | | 9.1 | | 22.3 | | 24.8 | | 2.1 | | 2.7 | | 8.4 | | 11.0 | |
| Arunachal Pradesh | 3.4 | | 4.2 | | 1.9 | | 1.2 | | 7.3 | | 10.1 | | 5.3 | | 3.0 | | 84.5 | | 76.3 | | 74.5 | | 69.6 | | 4.1 | | 8.4 | | 15.7 | | 22.3 | | 0.7 | | 1.0 | | 2.6 | | 4.0 | |
| Assam | 10.1 | | 16.4 | | 9.8 | | 6.0 | | 17.8 | | 21.9 | | 15.0 | | 9.4 | | 68.7 | | 55.6 | | 62.6 | | 68.9 | | 2.9 | | 5.6 | | 11.0 | | 13.4 | | 0.6 | | 0.5 | | 1.6 | | 2.3 | |
| Bihar | 16.7 | | 20.5 | | 10.5 | | 8.6 | | 23.6 | | 25.8 | | 17.4 | | 13.1 | | 56.3 | | 50.1 | | 60.0 | | 60.4 | | 3.1 | | 3.5 | | 10.1 | | 14.5 | | 0.4 | | 0.3 | | 2.0 | | 3.4 | |
| Chhattisgarh | 21.0 | | 20.2 | | 9.8 | | 8.2 | | 31.4 | | 26.1 | | 17.0 | | 13.9 | | 45.2 | | 51.0 | | 63.9 | | 64.8 | | 1.9 | | 2.2 | | 7.9 | | 10.5 | | 0.5 | | 0.4 | | 1.4 | | 2.6 | |
| Goa | 14.5 | | 16.0 | | 8.0 | | 5.2 | | 16.1 | | 13.7 | | 9.7 | | 4.9 | | 51.7 | | 53.2 | | 50.2 | | 53.1 | | 15.0 | | 13.9 | | 25.0 | | 27.8 | | 2.8 | | 3.2 | | 7.0 | | 9.1 | |
| Gujarat | 24.7 | | 21.7 | | 14.9 | | 12.3 | | 23.3 | | 20.5 | | 15.1 | | 13.4 | | 44.2 | | 46.3 | | 52.0 | | 54.5 | | 6.1 | | 8.8 | | 13.6 | | 14.5 | | 1.8 | | 2.7 | | 4.3 | | 5.3 | |
| Haryana | 12.4 | | 14.9 | | 5.7 | | 4.5 | | 19.0 | | 17.9 | | 8.4 | | 7.2 | | 58.7 | | 52.0 | | 64.0 | | 52.4 | | 8.1 | | 12.0 | | 17.2 | | 25.8 | | 1.8 | | 3.2 | | 4.7 | | 10.1 | |
| Himachal Pradesh | 14.3 | | 11.2 | | 4.5 | | 4.5 | | 16.6 | | 15.3 | | 8.1 | | 5.9 | | 57.6 | | 59.7 | | 56.1 | | 56.3 | | 9.8 | | 12.0 | | 23.9 | | 25.3 | | 1.8 | | 1.9 | | 7.6 | | 7.9 | |
| Jharkhand | 17.8 | | 20.0 | | 13.2 | | 9.3 | | 26.3 | | 27.6 | | 19.7 | | 16.5 | | 53.9 | | 50.7 | | 60.0 | | 64.0 | | 1.9 | | 1.6 | | 6.1 | | 8.4 | | 0.2 | | 0.1 | | 0.9 | | 1.8 | |
| Karnataka | 23.1 | | 17.6 | | 8.9 | | 6.6 | | 24.3 | | 21.3 | | 11.6 | | 9.1 | | 45.7 | | 51.2 | | 60.6 | | 55.4 | | 6.1 | | 8.3 | | 14.8 | | 21.7 | | 0.8 | | 1.5 | | 4.1 | | 7.1 | |
| Kerala | 7.1 | | 7.5 | | 2.4 | | 2.6 | | 13.4 | | 8.4 | | 4.8 | | 4.2 | | 59.6 | | 54.6 | | 57.3 | | 52.7 | | 16.4 | | 24.3 | | 28.7 | | 31.2 | | 3.5 | | 5.2 | | 6.8 | | 9.4 | |
| Madhya Pradesh | 15.5 | | 19.5 | | 11.0 | | 7.6 | | 23.1 | | 25.3 | | 17.4 | | 13.1 | | 58.3 | | 51.6 | | 60.7 | | 63.7 | | 2.7 | | 3.4 | | 9.0 | | 12.4 | | 0.4 | | 0.3 | | 1.9 | | 3.1 | |
| Maharashtra | 25.2 | | 19.9 | | 11.6 | | 9.1 | | 24.2 | | 23.5 | | 14.5 | | 11.5 | | 46.2 | | 48.2 | | 57.1 | | 58.6 | | 3.6 | | 7.0 | | 13.5 | | 16.4 | | 0.8 | | 1.3 | | 3.3 | | 4.4 | |
| Manipur | 5.9 | | 2.8 | | 1.6 | | 1.4 | | 13.5 | | 12.2 | | 5.6 | | 4.4 | | 72.2 | | 73.1 | | 67.4 | | 59.0 | | 7.3 | | 10.5 | | 21.0 | | 28.1 | | 1.0 | | 1.4 | | 4.4 | | 7.0 | |
| Meghalaya | 11.8 | | 4.7 | | 3.1 | | 2.2 | | 14.8 | | 9.0 | | 8.6 | | 6.4 | | 69.5 | | 80.9 | | 76.2 | | 79.8 | | 3.7 | | 4.3 | | 10.8 | | 10.5 | | 0.2 | | 1.1 | | 1.3 | | 1.1 | |
| Mizoram | 8.5 | | 6.0 | | 2.2 | | 1.2 | | 21.8 | | 12.8 | | 7.0 | | 4.2 | | 66.4 | | 76.1 | | 76.6 | | 75.7 | | 2.8 | | 4.8 | | 11.7 | | 16.2 | | 0.5 | | 0.3 | | 2.6 | | 2.6 | |
| Nagaland | 5.3 | | 3.4 | | 2.1 | | 2.1 | | 14.0 | | 12.8 | | 8.0 | | 4.9 | | 73.5 | | 78.9 | | 74.7 | | 78.1 | | 6.6 | | 4.5 | | 13.1 | | 13.4 | | 0.6 | | 0.4 | | 2.1 | | 1.5 | |
| Odisha | 21.8 | | 20.0 | | 11.1 | | 7.9 | | 28.5 | | 23.9 | | 15.5 | | 12.4 | | 46.3 | | 51.4 | | 58.2 | | 58.0 | | 3.0 | | 4.0 | | 12.5 | | 17.5 | | 0.4 | | 0.8 | | 2.7 | | 4.2 | |
| Punjab | 8.7 | | 5.9 | | 3.7 | | 3.7 | | 11.5 | | 9.7 | | 5.5 | | 5.5 | | 54.9 | | 53.0 | | 55.5 | | 46.9 | | 18.8 | | 22.3 | | 25.0 | | 28.9 | | 6.1 | | 9.0 | | 10.4 | | 15.0 | |
| Rajasthan | 15.4 | | 14.4 | | 10.2 | | 5.9 | | 24.4 | | 22.3 | | 15.3 | | 10.4 | | 56.6 | | 57.2 | | 61.4 | | 70.9 | | 3.0 | | 5.1 | | 10.7 | | 10.5 | | 0.6 | | 1.0 | | 2.4 | | 2.2 | |
| Sikkim | 2.4 | | 2.8 | | 1.1 | | 1.8 | | 8.4 | | 7.2 | | 3.5 | | 2.7 | | 74.8 | | 73.5 | | 68.6 | | 61.1 | | 11.7 | | 13.3 | | 22.1 | | 28.1 | | 2.7 | | 3.2 | | 4.7 | | 6.3 | |
| Tamil Nadu | 17.5 | | 14.4 | | 6.1 | | 4.5 | | 17.8 | | 16.7 | | 8.2 | | 7.1 | | 55.3 | | 54.1 | | 57.0 | | 49.4 | | 8.1 | | 12.4 | | 22.1 | | 27.3 | | 1.4 | | 2.5 | | 6.6 | | 11.8 | |
| Telangana | 21.6 | | 17.7 | | 11.3 | | 8.1 | | 25.0 | | 20.1 | | 14.0 | | 10.3 | | 47.1 | | 50.4 | | 54.0 | | 55.2 | | 5.9 | | 9.1 | | 16.4 | | 20.1 | | 0.5 | | 2.7 | | 4.3 | | 6.3 | |
| Tripura | 14.9 | | 17.1 | | 6.8 | | 5.3 | | 22.7 | | 20.8 | | 11.9 | | 9.0 | | 56.7 | | 56.0 | | 67.0 | | 65.2 | | 3.8 | | 5.8 | | 12.5 | | 17.7 | | 1.9 | | 0.3 | | 1.8 | | 2.8 | |
| Uttar Pradesh | 16.5 | | 15.5 | | 8.9 | | 5.6 | | 24.0 | | 22.0 | | 14.4 | | 10.1 | | 54.8 | | 54.7 | | 60.6 | | 61.5 | | 4.2 | | 6.6 | | 13.0 | | 17.8 | | 0.5 | | 1.1 | | 3.1 | | 5.0 | |
| Uttarakhand | 11.6 | | 11.8 | | 5.1 | | 3.5 | | 25.6 | | 19.5 | | 11.4 | | 7.9 | | 59.5 | | 58.0 | | 64.4 | | 58.8 | | 2.5 | | 8.5 | | 14.8 | | 23.3 | | 0.9 | | 2.2 | | 4.3 | | 6.5 | |
| West Bengal | 25.0 | | 20.6 | | 9.0 | | 5.4 | | 26.0 | | 24.1 | | 12.5 | | 8.6 | | 45.2 | | 49.1 | | 61.4 | | 62.9 | | 3.7 | | 5.7 | | 14.5 | | 19.0 | | 0.1 | | 0.5 | | 2.6 | | 4.0 | |
| NCT Delhi(UT) | 3.4 | | 7.5 | | 2.7 | | 2.0 | | 8.9 | | 8.2 | | 8.0 | | 2.6 | | 61.3 | | 62.6 | | 59.5 | | 46.5 | | 21.6 | | 16.3 | | 21.7 | | 31.3 | | 4.8 | | 5.4 | | 8.0 | | 17.7 | |
| Jammu&Kashmir (UT) | 11.6 | | 9.7 | | 3.5 | | 1.0 | | 18.7 | | 16.2 | | 7.4 | | 2.2 | | 60.7 | | 60.7 | | 60.6 | | 64.7 | | 7.3 | | 11.1 | | 21.1 | | 27.3 | | 1.6 | | 2.3 | | 7.3 | | 4.8 | |
| Ladakh(UT) |  | | 9.7 | | 1.4 | | 0.5 | |  | | 16.2 | | 7.4 | | 1.5 | |  | | 60.7 | | 74.7 | | 66.1 | |  | | 11.1 | | 14.5 | | 28.6 | |  | | 2.3 | | 2.0 | | 3.4 | |
| Andaman&Nicobar (UT) |  | |  | | 6.1 | | 1.8 | |  | |  | | 6.4 | | 3.6 | |  | |  | | 57.4 | | 55.0 | |  | |  | | 23.4 | | 27.0 | |  | |  | | 6.7 | | 12.6 | |
| Chandigarh(UT) |  | |  | | 5.0 | | 0.0 | |  | |  | | 25.0 | | 10.0 | |  | |  | | 55.0 | | 30.0 | |  | |  | | 5.0 | | 35.0 | |  | |  | | 10.0 | | 25.0 | |
| Dadra and Nagar Haveli and Diu(UT) |  | |  | | 13.5 | | 11.3 | |  | |  | | 16.8 | | 14.3 | |  | |  | | 56.6 | | 52.9 | |  | |  | | 10.7 | | 17.1 | |  | |  | | 2.5 | | 4.4 | |
| Lakshadweep(UT) |  | |  | | 7.9 | | 4.5 | |  | |  | | 8.0 | | 5.0 | |  | |  | | 45.3 | | 55.4 | |  | |  | | 23.5 | | 28.2 | |  | |  | | 15.4 | | 6.8 | |
| Puducherry(UT) |  | |  | | 3.1 | | 3.6 | |  | |  | | 5.1 | | 4.0 | |  | |  | | 54.1 | | 44.3 | |  | |  | | 28.2 | | 32.4 | |  | |  | | 9.5 | | 15.7 | |

B. Men, 2006-2021

|  | **Severely/Moderately Thin** | | | | | | | **Mildly Thin** | | | | | | | **Normal** | | | | | | | **Overweight** | | | | | | | **Obese** | | | | | | |
| --- | --- | --- | --- | --- | --- | --- | --- | --- | --- | --- | --- | --- | --- | --- | --- | --- | --- | --- | --- | --- | --- | --- | --- | --- | --- | --- | --- | --- | --- | --- | --- | --- | --- | --- | --- |
|  | <5 | 5-  9.9 | | 10-  14.9 | 15-  19.9 | | ≥20 | <5 | 5-  9.9 | | 10-  14.9 | 15-  19.9 | | ≥20 | <50 | 50-  54.9 | | 55-  59.9 | 60-  69.9 | | ≥70 | <5 | 5-  9.9 | | 10-  14.9 | 15-  19.9 | | ≥20 | <5 | 5-  9.9 | | 10-  14.9 | 15-  19.9 | | ≥20 |
|  |  | |  | | |  | |  | |  | | |  | |  | |  | | |  | |  | |  | | |  | |  | |  | | |  | |
|  | **2006** | | **2016** | | | **2021** | | **2006** | | **2016** | | | **2021** | | **2006** | | **2016** | | | **2021** | | **2006** | | **2016** | | | **2021** | | **2006** | | **2016** | | | **2021** | |
| **All-India** | 10.2 | | 4.9 | | | 3.7 | | 18.2 | | 9.8 | | | 7.0 | | 60.2 | | 63.1 | | | 62.7 | | 9.8 | | 18.6 | | | 22.1 | | 1.6 | | 3.5 | | | 4.5 | |
| Andhra Pradesh | 8.5 | | 3.6 | | | 3.3 | | 17.5 | | 7.2 | | | 7.7 | | 58.2 | | 52.8 | | | 53.3 | | 13.7 | | 27.7 | | | 29.0 | | 2.2 | | 8.7 | | | 6.7 | |
| Arunachal Pradesh | 3.8 | | 2.1 | | | 0.7 | | 10.1 | | 4.8 | | | 2.4 | | 76.6 | | 69.2 | | | 64.4 | | 9.3 | | 21.3 | | | 28.5 | | 0.2 | | 2.6 | | | 4.0 | |
| Assam | 11.2 | | 5.5 | | | 3.9 | | 21.1 | | 12.7 | | | 6.5 | | 61.7 | | 67.4 | | | 70.6 | | 5.5 | | 12.8 | | | 16.7 | | 0.6 | | 1.7 | | | 2.4 | |
| Bihar | 9.4 | | 4.8 | | | 4.4 | | 19.1 | | 13.0 | | | 9.1 | | 64.0 | | 65.8 | | | 67.3 | | 6.6 | | 14.7 | | | 17.0 | | 0.9 | | 1.8 | | | 2.2 | |
| Chhattisgarh | 8.7 | | 5.2 | | | 3.5 | | 23.2 | | 13.3 | | | 9.4 | | 61.8 | | 70.0 | | | 69.7 | | 5.4 | | 9.9 | | | 14.7 | | 0.8 | | 1.5 | | | 2.7 | |
| Goa | 8.7 | | 1.8 | | | 2.2 | | 11.1 | | 5.9 | | | 4.0 | | 61.8 | | 54.8 | | | 58.1 | | 16.0 | | 28.1 | | | 31.5 | | 2.4 | | 9.3 | | | 4.2 | |
| Gujarat | 13.3 | | 7.6 | | | 6.6 | | 17.8 | | 11.1 | | | 9.0 | | 54.3 | | 57.9 | | | 61.9 | | 12.1 | | 18.7 | | | 17.5 | | 2.5 | | 4.7 | | | 5.0 | |
| Haryana | 10.5 | | 2.2 | | | 2.6 | | 14.0 | | 5.2 | | | 6.2 | | 62.5 | | 69.4 | | | 57.1 | | 11.0 | | 20.2 | | | 27.1 | | 2.0 | | 2.9 | | | 7.0 | |
| Himachal Pradesh | 7.7 | | 3.3 | | | 1.3 | | 13.1 | | 7.9 | | | 5.3 | | 66.1 | | 63.3 | | | 57.3 | | 11.6 | | 21.7 | | | 30.4 | | 1.5 | | 3.8 | | | 5.7 | |
| Jharkhand | 10.9 | | 6.0 | | | 3.4 | | 21.7 | | 12.3 | | | 8.2 | | 61.4 | | 68.2 | | | 70.3 | | 5.4 | | 12.0 | | | 16.3 | | 0.5 | | 1.4 | | | 1.8 | |
| Karnataka | 10.4 | | 3.7 | | | 2.8 | | 17.2 | | 7.4 | | | 5.8 | | 59.4 | | 62.8 | | | 57.2 | | 11.1 | | 21.7 | | | 27.7 | | 2.0 | | 4.4 | | | 6.6 | |
| Kerala | 6.4 | | 1.3 | | | 1.6 | | 8.9 | | 3.4 | | | 3.1 | | 63.0 | | 63.4 | | | 52.9 | | 19.1 | | 28.2 | | | 34.9 | | 2.7 | | 3.8 | | | 7.5 | |
| Madhya Pradesh | 13.1 | | 7.3 | | | 5.0 | | 22.7 | | 14.8 | | | 9.7 | | 58.8 | | 65.0 | | | 67.1 | | 5.1 | | 11.1 | | | 15.5 | | 0.3 | | 1.9 | | | 2.7 | |
| Maharashtra | 10.6 | | 4.5 | | | 4.2 | | 16.2 | | 8.5 | | | 6.5 | | 58.6 | | 59.6 | | | 61.4 | | 12.6 | | 22.6 | | | 23.0 | | 1.9 | | 4.8 | | | 5.0 | |
| Manipur | 2.9 | | 1.6 | | | 1.2 | | 10.1 | | 6.6 | | | 3.5 | | 76.1 | | 68.1 | | | 60.5 | | 9.9 | | 20.6 | | | 30.8 | | 1.1 | | 3.1 | | | 4.0 | |
| Meghalaya | 1.7 | | 2.1 | | | 1.4 | | 8.4 | | 6.9 | | | 4.7 | | 81.9 | | 77.7 | | | 76.0 | | 7.9 | | 11.6 | | | 16.0 | | 0.1 | | 1.7 | | | 1.9 | |
| Mizoram | 0.9 | | 1.3 | | | 0.5 | | 5.7 | | 4.3 | | | 2.5 | | 80.1 | | 69.7 | | | 59.4 | | 12.8 | | 19.7 | | | 31.2 | | 0.6 | | 5.0 | | | 6.5 | |
| Nagaland | 2.7 | | 1.1 | | | 1.2 | | 8.1 | | 6.2 | | | 3.1 | | 82.2 | | 75.9 | | | 66.4 | | 6.5 | | 15.3 | | | 25.7 | | 0.6 | | 1.5 | | | 3.6 | |
| Odisha | 9.4 | | 4.9 | | | 3.7 | | 22.0 | | 11.3 | | | 8.3 | | 61.7 | | 64.8 | | | 64.0 | | 5.6 | | 16.4 | | | 19.8 | | 1.4 | | 2.6 | | | 4.2 | |
| Punjab | 4.5 | | 1.5 | | | 1.9 | | 9.6 | | 5.5 | | | 5.2 | | 59.0 | | 60.1 | | | 54.5 | | 22.2 | | 26.7 | | | 28.8 | | 4.7 | | 6.2 | | | 9.6 | |
| Rajasthan | 12.4 | | 6.1 | | | 2.4 | | 20.7 | | 10.8 | | | 6.1 | | 58.3 | | 66.9 | | | 72.4 | | 7.3 | | 13.5 | | | 17.7 | | 1.3 | | 2.7 | | | 1.4 | |
| Sikkim | 2.1 | | 0.5 | | | 0.7 | | 5.7 | | 1.4 | | | 0.6 | | 77.4 | | 57.3 | | | 60.0 | | 13.3 | | 34.2 | | | 33.3 | | 1.5 | | 6.6 | | | 5.5 | |
| Tamil Nadu | 8.4 | | 2.7 | | | 2.6 | | 12.7 | | 5.0 | | | 5.0 | | 62.3 | | 59.8 | | | 50.8 | | 14.3 | | 27.8 | | | 32.2 | | 2.3 | | 4.7 | | | 9.4 | |
| Telangana | 8.5 | | 4.7 | | | 4.2 | | 17.5 | | 10.6 | | | 6.4 | | 58.2 | | 57.9 | | | 53.2 | | 13.7 | | 21.4 | | | 28.3 | | 2.2 | | 5.4 | | | 8.0 | |
| Tripura | 14.8 | | 3.2 | | | 2.4 | | 20.2 | | 9.5 | | | 6.1 | | 59.5 | | 69.8 | | | 67.0 | | 5.5 | | 15.4 | | | 21.3 | | 0.0 | | 2.1 | | | 3.2 | |
| Uttar Pradesh | 11.2 | | 6.9 | | | 3.4 | | 21.3 | | 12.9 | | | 8.3 | | 58.1 | | 64.6 | | | 65.8 | | 8.0 | | 13.6 | | | 18.9 | | 1.4 | | 2.0 | | | 3.6 | |
| Uttarakhand | 9.0 | | 3.5 | | | 3.2 | | 13.8 | | 7.1 | | | 7.2 | | 66.4 | | 68.5 | | | 56.2 | | 9.3 | | 17.9 | | | 27.3 | | 1.5 | | 3.0 | | | 6.1 | |
| West Bengal | 11.2 | | 5.7 | | | 3.4 | | 20.2 | | 10.1 | | | 7.4 | | 62.2 | | 68.2 | | | 70.6 | | 6.0 | | 14.0 | | | 16.5 | | 0.5 | | 2.0 | | | 2.2 | |
| NCT Delhi(UT) | 2.9 | | 4.0 | | | 2.0 | | 8.5 | | 8.9 | | | 2.7 | | 67.5 | | 54.9 | | | 52.1 | | 18.1 | | 27.2 | | | 35.4 | | 2.9 | | 5.0 | | | 7.8 | |
| Jammu&Kashmir (UT) | 5.6 | | 1.4 | | | 0.6 | | 14.9 | | 6.6 | | | 1.7 | | 70.1 | | 66.9 | | | 61.1 | | 8.4 | | 21.1 | | | 32.0 | | 0.9 | | 3.9 | | | 4.5 | |
| Ladakh(UT) | 5.6 | | 0.4 | | | 0.0 | | 14.9 | | 5.8 | | | 1.1 | | 70.1 | | 73.0 | | | 56.6 | | 8.4 | | 18.7 | | | 38.8 | | 0.9 | | 2.1 | | | 3.5 | |
| Andaman&Nicobar (UT) |  | | 1.8 | | | 0.8 | |  | | 4.7 | | | 2.9 | |  | | 48.7 | | | 47.7 | |  | | 34.2 | | | 38.0 | |  | | 10.6 | | | 10.5 | |
| Chandigarh(UT) |  | | 3.0 | | | 2.9 | |  | | 10.5 | | | 2.2 | |  | | 49.3 | | | 54.1 | |  | | 31.8 | | | 30.9 | |  | | 5.4 | | | 10.0 | |
| Dadra and NagarHaveli and Diu(UT) |  | | 4.1 | | | 6.0 | |  | | 8.0 | | | 7.3 | |  | | 57.0 | | | 63.8 | |  | | 25.1 | | | 20.0 | |  | | 5.9 | | | 3.0 | |
| Lakshadweep(UT) |  | | 2.4 | | | 2.4 | |  | | 3.8 | | | 0.0 | |  | | 65.2 | | | 54.5 | |  | | 24.1 | | | 38.1 | |  | | 4.6 | | | 5.0 | |
| Puducherry(UT) |  | | 3.0 | | | 1.8 | |  | | 3.7 | | | 4.3 | |  | | 52.0 | | | 44.6 | |  | | 36.1 | | | 39.2 | |  | | 5.1 | | | 10.1 | |

**Note:** UT refers to Union Territories.

|  | **Severely/Moderately Thin** | | | | | | | **Mildly Thin** | | | | | | | **Normal** | | | | | | | **Overweight** | | | | | | | **Obese** | | | | | | |
| --- | --- | --- | --- | --- | --- | --- | --- | --- | --- | --- | --- | --- | --- | --- | --- | --- | --- | --- | --- | --- | --- | --- | --- | --- | --- | --- | --- | --- | --- | --- | --- | --- | --- | --- | --- |
|  | <5 | 5-  9.9 | | 10-  14.9 | 15-  19.9 | | ≥20 | <5 | 5-  9.9 | | 10-  14.9 | 15-  19.9 | | ≥20 | <50 | 50-  54.9 | | 55-  59.9 | 60-  69.9 | | ≥70 | <5 | 5-  9.9 | | 10-  14.9 | 15-  19.9 | | ≥20 | <5 | 5-  9.9 | | 10-  14.9 | 15-  19.9 | | ≥20 |
|  |  | |  | | |  | |  | |  | | |  | |  | |  | | |  | |  | |  | | |  | |  | |  | | |  | |
|  | **2006** | | **2016** | | | **2021** | | **2006** | | **2016** | | | **2021** | | **2006** | | **2016** | | | **2021** | | **2006** | | **2016** | | | **2021** | | **2006** | | **2016** | | | **2021** | |
| **Urban-India** | 7.8 | | 3.7 | | | 2.9 | | 12.2 | | 6.5 | | | 5.2 | | 60.5 | | 58.8 | | | 58.0 | | 16.6 | | 25.6 | | | 27.4 | | 3.0 | | 5.4 | | | 6.5 | |
| Andhra Pradesh | 3.8 | | 3.7 | | | 3.6 | | 10.7 | | 5.2 | | | 7.9 | | 59.1 | | 41.3 | | | 44.5 | | 20.2 | | 37.7 | | | 34.6 | | 4.0 | | 12.1 | | | 9.3 | |
| Arunachal Pradesh | 10.5 | | 1.1 | | | 1.5 | | 10.4 | | 5.3 | | | 2.1 | | 75.3 | | 63.0 | | | 59.8 | | 9.9 | | 28.1 | | | 31.3 | | 0.5 | | 2.4 | | | 5.3 | |
| Assam | 9.8 | | 4.7 | | | 4.4 | | 15.9 | | 9.0 | | | 3.8 | | 60.3 | | 60.5 | | | 62.4 | | 12.7 | | 21.7 | | | 24.2 | | 0.6 | | 4.1 | | | 5.2 | |
| Bihar | 7.7 | | 3.6 | | | 2.3 | | 14.5 | | 9.1 | | | 5.4 | | 64.3 | | 60.3 | | | 68.1 | | 9.5 | | 23.7 | | | 22.6 | | 1.9 | | 3.3 | | | 1.6 | |
| Chhattisgarh | 7.6 | | 5.7 | | | 1.9 | | 13.1 | | 10.3 | | | 5.5 | | 61.9 | | 62.6 | | | 65.9 | | 14.4 | | 17.6 | | | 21.8 | | 3.0 | | 3.8 | | | 4.8 | |
| Goa | 8.9 | | 1.3 | | | 2.4 | | 9.5 | | 5.3 | | | 2.5 | | 61.8 | | 52.7 | | | 59.7 | | 18.4 | | 30.2 | | | 31.2 | | 2.6 | | 10.5 | | | 4.2 | |
| Gujarat | 6.5 | | 5.1 | | | 4.7 | | 13.2 | | 7.4 | | | 6.4 | | 55.9 | | 56.4 | | | 60.0 | | 17.9 | | 24.0 | | | 21.6 | | 4.1 | | 7.0 | | | 7.3 | |
| Haryana | 3.6 | | 1.4 | | | 2.2 | | 10.6 | | 4.7 | | | 4.7 | | 59.8 | | 69.6 | | | 54.6 | | 18.7 | | 20.7 | | | 30.2 | | 4.5 | | 3.6 | | | 8.3 | |
| Himachal Pradesh | 10.8 | | 3.2 | | | 0.0 | | 8.3 | | 8.9 | | | 2.0 | | 66.5 | | 54.4 | | | 54.8 | | 18.7 | | 27.6 | | | 32.3 | | 2.9 | | 5.8 | | | 10.9 | |
| Jharkhand | 7.3 | | 3.6 | | | 1.9 | | 10.5 | | 8.2 | | | 5.2 | | 65.9 | | 63.6 | | | 67.9 | | 11.7 | | 22.0 | | | 22.6 | | 1.2 | | 2.6 | | | 2.4 | |
| Karnataka | 5.1 | | 3.3 | | | 2.4 | | 11.7 | | 5.6 | | | 3.4 | | 59.5 | | 57.6 | | | 51.3 | | 18.1 | | 27.6 | | | 33.8 | | 3.5 | | 5.9 | | | 9.0 | |
| Kerala | 9.9 | | 1.1 | | | 1.0 | | 8.5 | | 2.6 | | | 2.9 | | 61.4 | | 61.3 | | | 49.0 | | 21.9 | | 30.4 | | | 39.8 | | 3.1 | | 4.6 | | | 7.4 | |
| Madhya Pradesh | 8.0 | | 6.0 | | | 3.6 | | 14.5 | | 9.2 | | | 6.3 | | 63.3 | | 63.8 | | | 60.6 | | 11.7 | | 17.2 | | | 24.1 | | 0.6 | | 3.9 | | | 5.4 | |
| Maharashtra | 3.3 | | 2.8 | | | 3.6 | | 13.0 | | 5.5 | | | 6.5 | | 58.1 | | 55.3 | | | 57.1 | | 17.9 | | 29.7 | | | 26.3 | | 3.0 | | 6.8 | | | 6.5 | |
| Manipur | 0.5 | | 2.3 | | | 2.1 | | 8.2 | | 7.9 | | | 2.8 | | 71.1 | | 63.6 | | | 55.9 | | 15.6 | | 22.4 | | | 34.8 | | 1.7 | | 3.9 | | | 4.4 | |
| Meghalaya | 0.3 | | 2.5 | | | 1.3 | | 10.0 | | 6.2 | | | 3.0 | | 82.9 | | 68.4 | | | 54.1 | | 6.2 | | 20.5 | | | 36.0 | | 0.5 | | 2.3 | | | 5.5 | |
| Mizoram | 3.0 | | 1.8 | | | 0.1 | | 5.6 | | 3.2 | | | 1.0 | | 76.0 | | 61.2 | | | 54.6 | | 17.0 | | 26.0 | | | 36.0 | | 1.0 | | 7.9 | | | 8.3 | |
| Nagaland | 7.2 | | 0.9 | | | 1.7 | | 7.9 | | 6.1 | | | 2.5 | | 75.7 | | 71.4 | | | 59.6 | | 12.0 | | 20.0 | | | 31.4 | | 1.4 | | 1.6 | | | 4.7 | |
| Odisha | 4.5 | | 2.2 | | | 2.1 | | 13.0 | | 6.9 | | | 5.1 | | 63.2 | | 55.5 | | | 56.9 | | 12.0 | | 30.8 | | | 27.6 | | 4.6 | | 4.6 | | | 8.3 | |
| Punjab | 9.6 | | 1.3 | | | 1.8 | | 9.2 | | 5.2 | | | 4.0 | | 55.0 | | 56.7 | | | 52.1 | | 24.9 | | 31.1 | | | 31.2 | | 6.4 | | 5.7 | | | 11.0 | |
| Rajasthan | 2.5 | | 4.0 | | | 1.0 | | 13.3 | | 7.3 | | | 4.3 | | 58.9 | | 64.9 | | | 70.1 | | 15.0 | | 18.6 | | | 22.4 | | 3.2 | | 5.3 | | | 2.2 | |
| Sikkim | 7.2 | | 0.4 | | | 0.0 | | 4.0 | | 0.4 | | | 0.0 | | 73.6 | | 52.8 | | | 60.3 | | 18.5 | | 36.1 | | | 28.7 | | 1.4 | | 10.3 | | | 10.9 | |
| Tamil Nadu | 9.7 | | 2.3 | | | 2.3 | | 10.6 | | 4.5 | | | 4.8 | | 61.3 | | 58.4 | | | 44.1 | | 17.7 | | 29.7 | | | 35.4 | | 3.2 | | 5.1 | | | 13.3 | |
| Telangana | 3.8 | | 3.5 | | | 2.9 | | 10.7 | | 8.6 | | | 4.9 | | 59.1 | | 53.6 | | | 44.9 | | 20.2 | | 26.5 | | | 35.1 | | 4.0 | | 7.8 | | | 12.3 | |
| Tripura | 10.4 | | 1.8 | | | 3.2 | | 20.1 | | 7.3 | | | 7.1 | | 54.9 | | 70.0 | | | 59.8 | | 14.6 | | 18.9 | | | 25.7 | | 0.0 | | 1.9 | | | 4.3 | |
| Uttar Pradesh | 8.5 | | 5.1 | | | 2.7 | | 14.8 | | 7.6 | | | 5.4 | | 57.5 | | 62.2 | | | 62.2 | | 16.0 | | 21.5 | | | 24.1 | | 3.2 | | 3.7 | | | 5.7 | |
| Uttarakhand | 7.6 | | 4.2 | | | 2.3 | | 7.2 | | 5.2 | | | 9.2 | | 68.1 | | 63.5 | | | 47.0 | | 15.2 | | 22.9 | | | 33.8 | | 1.9 | | 4.3 | | | 7.6 | |
| West Bengal | 8.6 | | 6.7 | | | 2.3 | | 10.9 | | 8.4 | | | 4.6 | | 66.2 | | 62.1 | | | 71.6 | | 13.0 | | 19.7 | | | 19.1 | | 1.3 | | 3.0 | | | 2.3 | |
| NCT Delhi(UT) | 2.7 | | 4.0 | | | 2.0 | | 8.6 | | 9.0 | | | 2.7 | | 66.7 | | 55.2 | | | 52.2 | | 18.8 | | 26.8 | | | 35.4 | | 3.2 | | 5.0 | | | 7.7 | |
| Jammu&Kashmir (UT) | 4.4 | | 0.6 | | | 0.5 | | 9.9 | | 4.3 | | | 0.5 | | 68.5 | | 58.9 | | | 51.0 | | 15.0 | | 29.6 | | | 39.8 | | 2.2 | | 6.6 | | | 8.2 | |
| Ladakh(UT) | 4.4 | | 0.5 | | | 0.0 | | 9.9 | | 9.6 | | | 1.4 | | 68.5 | | 63.9 | | | 48.8 | | 15.0 | | 22.6 | | | 43.4 | | 2.2 | | 3.5 | | | 6.5 | |
| Andaman&Nicobar (UT) |  | | 2.5 | | | 1.1 | |  | | 5.6 | | | 6.2 | |  | | 46.4 | | | 52.7 | |  | | 32.9 | | | 36.5 | |  | | 12.6 | | | 3.5 | |
| Chandigarh(UT) |  | | 3.3 | | | 2.9 | |  | | 6.4 | | | 2.2 | |  | | 50.6 | | | 54.1 | |  | | 33.8 | | | 30.9 | |  | | 6.0 | | | 10.0 | |
| Dadra and NagarHaveli and Diu(UT) |  | | 2.6 | | | 6.8 | |  | | 6.0 | | | 6.5 | |  | | 54.1 | | | 56.7 | |  | | 30.3 | | | 25.1 | |  | | 7.0 | | | 4.9 | |
| Lakshadweep(UT) |  | | 3.0 | | | 0.0 | |  | | 3.9 | | | 0.0 | |  | | 64.2 | | | 55.7 | |  | | 26.4 | | | 40.8 | |  | | 2.4 | | | 3.5 | |
| Puducherry(UT) |  | | 3.3 | | | 1.5 | |  | | 4.0 | | | 3.2 | |  | | 48.9 | | | 45.0 | |  | | 38.4 | | | 41.6 | |  | | 5.4 | | | 8.7 | |

|  | **Severely/Moderately Thin** | | | | | | | **Mildly Thin** | | | | | | | **Normal** | | | | | | | **Overweight** | | | | | | | **Obese** | | | | | | |
| --- | --- | --- | --- | --- | --- | --- | --- | --- | --- | --- | --- | --- | --- | --- | --- | --- | --- | --- | --- | --- | --- | --- | --- | --- | --- | --- | --- | --- | --- | --- | --- | --- | --- | --- | --- |
|  | <5 | 5-  9.9 | | 10-  14.9 | 15-  19.9 | | ≥20 | <5 | 5-  9.9 | | 10-  14.9 | 15-  19.9 | | ≥20 | <50 | 50-  54.9 | | 55-  59.9 | 60-  69.9 | | ≥70 | <5 | 5-  9.9 | | 10-  14.9 | 15-  19.9 | | ≥20 | <5 | 5-  9.9 | | 10-  14.9 | 15-  19.9 | | ≥20 |
|  |  | |  | | |  | |  | |  | | |  | |  | |  | | |  | |  | |  | | |  | |  | |  | | |  | |
|  | **2006** | | **2016** | | | **2021** | | **2006** | | **2016** | | | **2021** | | **2006** | | **2016** | | | **2021** | | **2006** | | **2016** | | | **2021** | | **2006** | | **2016** | | | **2021** | |
| **Rural-India** | 11.6 | | 5.6 | | | 4.1 | | 21.5 | | 11.7 | | | 8.0 | | 60.0 | | 65.6 | | | 65.0 | | 6.2 | | 14.6 | | | 19.4 | | 0.8 | | 2.4 | | | 3.5 | |
| Andhra Pradesh | 9.8 | | 3.5 | | | 3.1 | | 21.0 | | 8.1 | | | 7.6 | | 57.7 | | 57.9 | | | 57.1 | | 10.3 | | 23.3 | | | 26.6 | | 1.2 | | 7.2 | | | 5.6 | |
| Arunachal Pradesh | 3.8 | | 2.4 | | | 0.6 | | 10.0 | | 4.6 | | | 2.4 | | 77.2 | | 71.2 | | | 65.2 | | 9.0 | | 19.1 | | | 28.0 | | 0.0 | | 2.7 | | | 3.8 | |
| Assam | 11.4 | | 5.6 | | | 3.8 | | 22.4 | | 13.5 | | | 7.0 | | 62.0 | | 68.8 | | | 72.1 | | 3.6 | | 10.9 | | | 15.2 | | 0.6 | | 1.2 | | | 1.8 | |
| Bihar | 9.3 | | 5.0 | | | 4.9 | | 20.4 | | 13.8 | | | 10.1 | | 64.0 | | 67.0 | | | 67.0 | | 5.7 | | 12.7 | | | 15.5 | | 0.6 | | 1.5 | | | 2.4 | |
| Chhattisgarh | 9.1 | | 5.1 | | | 4.0 | | 26.5 | | 14.3 | | | 10.5 | | 61.8 | | 72.5 | | | 70.8 | | 2.5 | | 7.4 | | | 12.6 | | 0.1 | | 0.8 | | | 2.1 | |
| Goa | 10.0 | | 2.8 | | | 2.0 | | 12.9 | | 6.7 | | | 6.7 | | 61.8 | | 58.3 | | | 55.2 | | 13.1 | | 24.6 | | | 32.0 | | 2.2 | | 7.5 | | | 4.1 | |
| Gujarat | 16.5 | | 9.6 | | | 8.1 | | 21.2 | | 14.1 | | | 10.9 | | 53.2 | | 59.1 | | | 63.4 | | 7.7 | | 14.4 | | | 14.4 | | 1.4 | | 2.7 | | | 3.2 | |
| Haryana | 12.4 | | 2.8 | | | 2.8 | | 15.7 | | 5.6 | | | 7.0 | | 63.8 | | 69.2 | | | 58.3 | | 7.3 | | 19.9 | | | 25.6 | | 0.8 | | 2.4 | | | 6.4 | |
| Himachal Pradesh | 8.4 | | 3.3 | | | 1.5 | | 13.9 | | 7.7 | | | 5.9 | | 66.0 | | 64.9 | | | 57.7 | | 10.5 | | 20.7 | | | 30.1 | | 1.2 | | 3.4 | | | 4.8 | |
| Jharkhand | 10.9 | | 7.0 | | | 4.0 | | 26.7 | | 14.0 | | | 9.3 | | 59.5 | | 70.1 | | | 71.2 | | 2.7 | | 8.1 | | | 13.9 | | 0.2 | | 0.9 | | | 1.6 | |
| Karnataka | 12.5 | | 4.1 | | | 3.1 | | 21.0 | | 8.8 | | | 7.2 | | 59.4 | | 66.6 | | | 60.7 | | 6.2 | | 17.2 | | | 23.9 | | 0.9 | | 3.3 | | | 5.1 | |
| Kerala | 7.1 | | 1.5 | | | 2.1 | | 9.1 | | 4.0 | | | 3.3 | | 63.9 | | 65.1 | | | 56.1 | | 17.5 | | 26.2 | | | 30.7 | | 2.4 | | 3.1 | | | 7.7 | |
| Madhya Pradesh | 14.5 | | 7.8 | | | 5.4 | | 26.1 | | 17.4 | | | 10.8 | | 56.9 | | 65.6 | | | 69.2 | | 2.3 | | 8.3 | | | 12.7 | | 0.2 | | 0.9 | | | 1.9 | |
| Maharashtra | 13.3 | | 6.0 | | | 4.6 | | 19.6 | | 11.3 | | | 6.5 | | 59.1 | | 63.4 | | | 64.6 | | 7.1 | | 16.3 | | | 20.5 | | 0.8 | | 3.0 | | | 3.8 | |
| Manipur | 2.6 | | 1.2 | | | 0.6 | | 11.0 | | 5.8 | | | 3.9 | | 78.6 | | 70.9 | | | 63.9 | | 7.0 | | 19.5 | | | 27.8 | | 0.7 | | 2.6 | | | 3.7 | |
| Meghalaya | 2.0 | | 2.0 | | | 1.4 | | 7.8 | | 7.1 | | | 5.1 | | 81.6 | | 80.1 | | | 80.8 | | 8.5 | | 9.3 | | | 11.7 | | 0.0 | | 1.5 | | | 1.1 | |
| Mizoram | 1.5 | | 0.6 | | | 0.9 | | 5.9 | | 5.7 | | | 4.3 | | 84.8 | | 81.2 | | | 65.2 | | 7.8 | | 11.3 | | | 25.3 | | 0.0 | | 1.2 | | | 4.3 | |
| Nagaland | 2.5 | | 1.3 | | | 0.9 | | 8.1 | | 6.2 | | | 3.4 | | 85.1 | | 78.4 | | | 70.4 | | 4.1 | | 12.7 | | | 22.3 | | 0.2 | | 1.5 | | | 3.0 | |
| Odisha | 9.9 | | 5.5 | | | 4.1 | | 24.0 | | 12.4 | | | 9.1 | | 61.3 | | 67.1 | | | 65.7 | | 4.1 | | 12.9 | | | 17.9 | | 0.7 | | 2.1 | | | 3.3 | |
| Punjab | 4.4 | | 1.7 | | | 1.9 | | 9.9 | | 5.7 | | | 6.0 | | 62.2 | | 62.3 | | | 56.1 | | 20.0 | | 23.8 | | | 27.2 | | 3.4 | | 6.5 | | | 8.7 | |
| Rajasthan | 13.7 | | 6.9 | | | 2.9 | | 24.2 | | 12.3 | | | 6.7 | | 58.1 | | 67.8 | | | 73.3 | | 3.7 | | 11.4 | | | 16.0 | | 0.4 | | 1.6 | | | 1.1 | |
| Sikkim | 2.0 | | 0.6 | | | 1.1 | | 6.2 | | 2.2 | | | 0.9 | | 78.5 | | 60.7 | | | 59.8 | | 11.9 | | 32.8 | | | 36.0 | | 1.5 | | 3.8 | | | 2.1 | |
| Tamil Nadu | 9.7 | | 3.2 | | | 2.8 | | 14.8 | | 5.6 | | | 5.1 | | 63.3 | | 61.3 | | | 56.2 | | 10.9 | | 25.7 | | | 29.6 | | 1.4 | | 4.3 | | | 6.3 | |
| Telangana | 9.8 | | 5.5 | | | 4.8 | | 21.0 | | 12.1 | | | 7.1 | | 57.7 | | 61.1 | | | 57.1 | | 10.3 | | 17.7 | | | 25.1 | | 1.2 | | 3.6 | | | 5.9 | |
| Tripura | 15.7 | | 3.8 | | | 2.1 | | 20.2 | | 10.5 | | | 5.7 | | 60.4 | | 69.7 | | | 70.2 | | 3.7 | | 13.8 | | | 19.3 | | 0.0 | | 2.2 | | | 2.7 | |
| Uttar Pradesh | 12.4 | | 7.8 | | | 3.7 | | 23.9 | | 15.3 | | | 9.2 | | 58.4 | | 65.7 | | | 66.9 | | 4.7 | | 10.1 | | | 17.2 | | 0.7 | | 1.2 | | | 3.0 | |
| Uttarakhand | 9.8 | | 3.0 | | | 3.6 | | 17.3 | | 8.3 | | | 6.3 | | 65.6 | | 71.7 | | | 60.1 | | 6.1 | | 14.7 | | | 24.5 | | 1.2 | | 2.2 | | | 5.4 | |
| West Bengal | 12.5 | | 5.2 | | | 3.9 | | 24.9 | | 10.8 | | | 8.7 | | 60.1 | | 71.0 | | | 70.0 | | 2.4 | | 11.3 | | | 15.2 | | 0.1 | | 1.6 | | | 2.1 | |
| NCT Delhi(UT) | 4.8 | | 0.0 | | | 0.0 | | 8.1 | | 0.0 | | | 0.0 | | 75.8 | | 11.0 | | | 46.3 | | 11.3 | | 89.0 | | | 41.1 | | 0.0 | | 0.0 | | | 12.6 | |
| Jammu&Kashmir (UT) | 6.1 | | 1.8 | | | 0.7 | | 17.3 | | 7.8 | | | 2.2 | | 70.9 | | 71.0 | | | 64.9 | | 5.4 | | 16.8 | | | 29.1 | | 0.4 | | 2.6 | | | 3.2 | |
| Ladakh(UT) | 6.1 | | 0.4 | | | 0.0 | | 17.3 | | 3.6 | | | 1.0 | | 70.9 | | 78.3 | | | 58.5 | | 5.4 | | 16.4 | | | 37.8 | | 0.4 | | 1.4 | | | 2.8 | |
| Andaman&Nicobar (UT) |  | | 1.3 | | | 0.6 | |  | | 4.1 | | | 0.6 | |  | | 50.4 | | | 44.2 | |  | | 35.1 | | | 39.1 | |  | | 9.2 | | | 15.5 | |
| Chandigarh(UT) |  | | 0.0 | | | 0.0 | |  | | 50.0 | | | 0.0 | |  | | 37.5 | | | 0.0 | |  | | 12.5 | | | 0.0 | |  | | 0.0 | | | 0.0 | |
| Dadra and NagarHaveli and Diu(UT) |  | | 6.7 | | | 5.4 | |  | | 11.5 | | | 7.9 | |  | | 62.1 | | | 69.5 | |  | | 15.7 | | | 15.9 | |  | | 4.0 | | | 1.4 | |
| Lakshadweep(UT) |  | | 0.0 | | | 9.4 | |  | | 3.3 | | | 0.0 | |  | | 68.6 | | | 51.2 | |  | | 15.7 | | | 30.0 | |  | | 12.4 | | | 9.4 | |
| Puducherry(UT) |  | | 2.5 | | | 2.6 | |  | | 3.1 | | | 6.8 | |  | | 58.5 | | | 43.4 | |  | | 31.4 | | | 33.3 | |  | | 4.5 | | | 13.9 | |

**Figure S4. SAC in the prevalence of BMI across States/Union Territories, 1999-2021.**

**A. Women**

| <-1.00 | -1.00 to-0.76 | -0.75 to -0.51 | -0.50 to -0.26 | -0.25 to -0.01 | 0.00 to 0.25 | 0.26 to 0.50 | 0.51 to 0.75 | 0.76 to 1.00 | >1.00 |
| --- | --- | --- | --- | --- | --- | --- | --- | --- | --- |

|  | **Severely/Moderately Thin** | | | **Mildly Thin** | | | **Normal** | | | **Overweight** | | | **Obese** | | |
| --- | --- | --- | --- | --- | --- | --- | --- | --- | --- | --- | --- | --- | --- | --- | --- |
|  | 1999-  2006 | 2006-2016 | 2016-  2021 | 1999-  2006 | 2006-  2016 | 2016-2021 | 1999-2006 | 2006-2016 | 2016-2021 | 1999-2006 | 2006-2016 | 2016-2021 | 1999-2006 | 2006-2016 | 2016-2021 |
| **All-India** | -0.19 | -0.70 | -0.38 | -0.24 | -0.70 | -0.50 | -0.10 | 0.50 | 0.15 | 0.39 | 0.64 | 0.46 | 0.15 | 0.26 | 0.29 |
| Andhra Pradesh | -0.38 | -0.87 | -0.18 | -0.37 | -0.80 | -0.42 | -0.05 | -0.19 | -0.01 | 0.47 | 1.20 | 0.26 | 0.34 | 0.66 | 0.35 |
| Arunachal Pradesh | 0.23 | -0.29 | -0.13 | 0.35 | -0.52 | -0.40 | -1.38 | -0.23 | -0.66 | 0.69 | 0.85 | 0.99 | 0.12 | 0.20 | 0.20 |
| Assam | 0.77 | -0.60 | -0.69 | 0.42 | -0.63 | -0.98 | -1.88 | 0.63 | 1.22 | 0.65 | 0.46 | 0.33 | 0.05 | 0.13 | 0.11 |
| Bihar | 0.35 | -0.89 | -0.39 | 0.11 | -0.77 | -0.82 | -0.68 | 0.78 | 0.10 | 0.18 | 0.69 | 0.85 | 0.04 | 0.19 | 0.26 |
| Chhattisgarh | -0.33 | -0.95 | -0.28 | -0.75 | -0.82 | -0.53 | 0.75 | 1.04 | 0.35 | 0.25 | 0.60 | 0.25 | 0.07 | 0.12 | 0.20 |
| Goa | -0.04 | -0.77 | -0.11 | -0.36 | -0.57 | -0.17 | 0.19 | -0.14 | -0.12 | 0.08 | 0.93 | 0.44 | 0.12 | 0.55 | -0.04 |
| Gujarat | -0.34 | -0.55 | -0.31 | -0.27 | -0.42 | -0.23 | 0.15 | 0.20 | 0.84 | 0.34 | 0.49 | -0.23 | 0.11 | 0.27 | -0.06 |
| Haryana | 0.29 | -0.78 | -0.12 | -0.04 | -0.81 | -0.20 | -0.79 | 1.28 | -2.45 | 0.37 | 0.27 | 1.60 | 0.17 | 0.03 | 1.17 |
| Himachal Pradesh | -0.42 | -0.62 | 0.01 | -0.25 | -0.67 | -0.45 | 0.30 | -0.36 | 0.05 | 0.35 | 1.11 | 0.29 | 0.02 | 0.54 | 0.10 |
| Jharkhand | 0.11 | -0.61 | -0.70 | -0.08 | -0.69 | -0.55 | -0.48 | 0.73 | 0.92 | 0.34 | 0.44 | 0.25 | 0.11 | 0.14 | 0.08 |
| Karnataka | -0.50 | -0.74 | -0.40 | -0.43 | -0.75 | -0.41 | 0.46 | 0.67 | -0.73 | 0.28 | 0.52 | 1.11 | 0.19 | 0.29 | 0.42 |
| Kerala | 0.02 | -0.48 | 0.06 | -0.62 | -0.37 | -0.08 | -0.93 | 0.41 | -1.34 | 1.29 | 0.28 | 0.58 | 0.23 | 0.16 | 0.78 |
| Madhya Pradesh | 0.50 | -0.82 | -0.56 | 0.24 | -0.77 | -0.64 | -0.95 | 0.86 | 0.52 | 0.18 | 0.52 | 0.52 | 0.03 | 0.21 | 0.15 |
| Maharashtra | -0.69 | -0.66 | -0.29 | -0.24 | -0.65 | -0.35 | 0.30 | 0.36 | 0.75 | 0.46 | 0.69 | -0.13 | 0.17 | 0.26 | 0.02 |
| Manipur | -0.49 | -0.13 | -0.04 | -0.33 | -0.58 | -0.29 | 0.16 | -0.71 | -1.38 | 0.51 | 1.03 | 1.26 | 0.15 | 0.39 | 0.46 |
| Meghalaya | -0.87 | -0.18 | -0.13 | -0.71 | -0.11 | -0.40 | 1.48 | -0.50 | 0.68 | 0.13 | 0.68 | -0.06 | -0.03 | 0.10 | -0.10 |
| Mizoram | -0.27 | -0.28 | -0.09 | -0.97 | -0.41 | -0.52 | 0.31 | -0.51 | 0.08 | 0.80 | 0.92 | 0.37 | 0.14 | 0.28 | 0.17 |
| Nagaland | -0.32 | -0.13 | -0.01 | -0.01 | -0.42 | -0.60 | 0.52 | -0.53 | 1.02 | -0.20 | 0.86 | -0.19 | 0.01 | 0.22 | -0.22 |
| Odisha | -0.39 | -0.84 | -0.56 | -0.71 | -0.81 | -0.58 | 0.67 | 0.52 | -0.23 | 0.32 | 0.86 | 1.03 | 0.10 | 0.27 | 0.34 |
| Punjab | -0.22 | -0.25 | 0.10 | -0.08 | -0.43 | 0.05 | -0.28 | 0.63 | -2.24 | 0.34 | 0.09 | 0.98 | 0.24 | -0.05 | 1.10 |
| Rajasthan | -0.13 | -0.44 | -0.72 | -0.35 | -0.68 | -0.84 | 0.04 | 0.48 | 1.85 | 0.38 | 0.45 | -0.15 | 0.06 | 0.18 | -0.14 |
| Sikkim | -0.01 | -0.15 | 0.10 | -0.26 | -0.31 | -0.09 | -0.04 | -0.79 | -1.50 | 0.15 | 0.98 | 1.25 | 0.16 | 0.27 | 0.24 |
| Tamil Nadu | -0.30 | -0.69 | -0.18 | -0.21 | -0.75 | -0.15 | -0.71 | 0.32 | -1.62 | 0.79 | 0.75 | 0.72 | 0.43 | 0.36 | 1.23 |
| Telangana | -0.35 | -0.59 | -0.36 | -0.45 | -0.62 | -0.36 | 0.29 | -0.15 | 0.45 | 0.16 | 0.88 | 0.23 | 0.37 | 0.48 | 0.04 |
| Tripura | 0.33 | -0.95 | -0.31 | -0.30 | -0.86 | -0.49 | 0.04 | 0.85 | -0.41 | 0.07 | 0.77 | 0.87 | -0.14 | 0.18 | 0.33 |
| Uttar Pradesh | -0.08 | -0.66 | -0.56 | -0.35 | -0.70 | -0.71 | -0.11 | 0.47 | 0.17 | 0.43 | 0.63 | 0.79 | 0.10 | 0.26 | 0.31 |
| Uttarakhand | 0.08 | -0.49 | -0.33 | -0.61 | -0.65 | -0.61 | 0.01 | 0.29 | -1.15 | 0.61 | 0.58 | 1.51 | -0.09 | 0.27 | 0.58 |
| West Bengal | -0.69 | -0.94 | -0.54 | -0.44 | -0.93 | -0.71 | 0.47 | 0.96 | 0.63 | 0.51 | 0.72 | 0.45 | 0.15 | 0.19 | 0.17 |
| NCT Delhi (UT) | 0.03 | -0.02 | -0.26 | -0.12 | -0.13 | -0.47 | 0.60 | -0.63 | -0.93 | -0.52 | 0.54 | 0.76 | 0.00 | 0.24 | 0.90 |
| Jammu&Kashmir (UT) | -0.28 | -0.52 | -0.38 | -0.39 | -0.74 | -0.87 | -0.17 | -0.12 | 1.30 | 0.68 | 0.90 | 0.63 | 0.15 | 0.47 | -0.68 |
| Ladakh (UT) |  | -0.67 | -0.17 |  | -0.72 | -1.02 |  | 1.54 | -1.53 |  | 0.01 | 2.50 |  | -0.17 | 0.21 |
| Andaman&Nicobar (UT) |  |  | -0.55 |  |  | -0.31 |  |  | -0.47 |  |  | 0.29 |  |  | 1.04 |
| Chandigarh (UT) |  |  | -0.18 |  |  | -0.02 |  |  | -0.23 |  |  | 0.08 |  |  | 0.36 |
| Dadra and Nagar Haveli and Diu (UT) |  |  | 0.22 |  |  | 0.05 |  |  | -0.91 |  |  | 0.40 |  |  | 0.24 |
| Lakshadweep (UT) |  |  | -0.58 |  |  | -0.62 |  |  | 2.62 |  |  | 0.20 |  |  | -1.61 |
| Puducherry (UT) |  |  | -0.04 |  |  | -0.07 |  |  | -1.98 |  |  | 0.36 |  |  | 1.73 |

|  | **Severely/Moderately Thin** | | | **Mildly Thin** | | | **Normal** | | | **Overweight** | | | **Obese** | | |
| --- | --- | --- | --- | --- | --- | --- | --- | --- | --- | --- | --- | --- | --- | --- | --- |
|  | 1999-  2006 | 2006-2016 | 2016-  2021 | 1999-  2006 | 2006-  2016 | 2016-2021 | 1999-2006 | 2006-2016 | 2016-2021 | 1999-2006 | 2006-2016 | 2016-2021 | 1999-2006 | 2006-2016 | 2016-2021 |
| **Urban-India** | -0.06 | -0.47 | -0.18 | -0.13 | -0.46 | -0.27 | -0.84 | 0.13 | 0.10 | 0.65 | 0.49 | 0.09 | 0.17 | 0.32 | 0.26 |
| Andhra Pradesh | -0.34 | -0.47 | 0.03 | -0.13 | -0.49 | -0.03 | -4.19 | -0.95 | 0.32 | 0.70 | 1.00 | -0.28 | 0.74 | 0.91 | -0.04 |
| Arunachal Pradesh | 0.59 | -0.47 | -0.05 | 0.06 | -0.64 | -0.33 | 0.02 | -0.41 | -0.21 | 1.08 | 1.21 | 0.41 | 0.37 | 0.31 | 0.18 |
| Assam | 0.59 | -0.38 | -0.34 | 0.07 | -0.50 | -0.21 | -0.31 | 0.25 | 1.08 | 0.37 | 0.33 | -0.38 | 0.20 | 0.30 | -0.15 |
| Bihar | -0.41 | -0.44 | -0.29 | -0.45 | -0.54 | -0.36 | 0.83 | -0.35 | 0.30 | 0.70 | 0.97 | 0.30 | 0.18 | 0.36 | 0.04 |
| Chhattisgarh | -0.95 | -0.60 | -0.19 | -0.34 | -0.46 | -0.31 | 0.65 | 0.30 | 0.83 | 0.09 | 0.60 | -0.44 | 0.29 | 0.16 | 0.11 |
| Goa | -0.10 | -0.69 | 0.15 | -0.24 | -0.63 | 0.31 | 0.33 | -0.04 | -0.57 | 0.29 | 0.75 | 0.40 | 0.03 | 0.60 | -0.29 |
| Gujarat | -0.24 | -0.36 | -0.12 | -0.10 | -0.25 | -0.15 | -0.27 | -0.26 | 1.26 | -0.21 | 0.47 | -0.65 | 0.08 | 0.39 | -0.34 |
| Haryana | 0.22 | -0.41 | 0.00 | 0.31 | -0.43 | -0.22 | -0.18 | 1.48 | -2.69 | 0.32 | -0.36 | 1.49 | 0.03 | -0.28 | 1.42 |
| Himachal Pradesh | -0.03 | -0.22 | 0.06 | -0.65 | -0.31 | -0.37 | -1.06 | -0.42 | 0.38 | 1.09 | 0.55 | -0.11 | -0.12 | 0.39 | 0.04 |
| Jharkhand | -0.21 | -0.44 | -0.56 | -0.33 | -0.42 | -0.49 | 0.81 | 0.21 | 1.17 | 0.06 | 0.39 | -0.04 | 0.37 | 0.27 | -0.07 |
| Karnataka | 0.05 | -0.49 | -0.37 | -0.31 | -0.34 | -0.37 | -1.70 | 0.36 | -0.44 | 1.41 | 0.21 | 0.88 | 0.26 | 0.26 | 0.31 |
| Kerala | 0.01 | -0.41 | 0.10 | -0.22 | -0.34 | -0.03 | -0.64 | 0.66 | -1.79 | 0.42 | -0.04 | 0.67 | 0.14 | 0.13 | 1.06 |
| Madhya Pradesh | 0.30 | -0.70 | -0.37 | 0.06 | -0.62 | -0.31 | -0.26 | 0.78 | 0.22 | 0.15 | 0.30 | 0.39 | 0.19 | 0.25 | 0.07 |
| Maharashtra | -0.38 | -0.48 | -0.13 | -0.15 | -0.40 | -0.18 | -1.78 | -0.17 | 1.16 | 0.62 | 0.72 | -0.75 | 0.15 | 0.33 | -0.11 |
| Manipur | -0.55 | -0.15 | -0.05 | -0.62 | -0.38 | -0.39 | -0.01 | -0.77 | -0.99 | 0.08 | 0.85 | 1.03 | 0.34 | 0.45 | 0.40 |
| Meghalaya | -0.30 | -0.23 | 0.00 | -0.31 | -0.31 | -0.27 | -0.27 | -0.65 | 0.41 | 1.13 | 0.81 | 0.12 | -0.65 | 0.38 | -0.26 |
| Mizoram | -0.16 | -0.20 | -0.02 | -0.61 | -0.28 | -0.51 | 0.04 | -0.88 | 0.13 | -0.16 | 1.05 | 0.08 | 0.25 | 0.32 | 0.32 |
| Nagaland | -0.52 | -0.16 | -0.01 | 0.68 | -0.24 | -0.58 | 2.41 | -0.48 | 1.47 | 0.81 | 0.63 | -0.53 | 0.09 | 0.25 | -0.34 |
| Odisha | -0.63 | -0.63 | -0.16 | -0.36 | -0.67 | -0.42 | 1.12 | -0.25 | -0.96 | -0.17 | 0.89 | 1.07 | 0.21 | 0.65 | 0.47 |
| Punjab | 0.21 | -0.28 | 0.24 | 0.34 | -0.43 | 0.09 | -1.01 | 1.25 | -3.08 | 0.34 | -0.19 | 1.34 | -0.30 | -0.35 | 1.41 |
| Rajasthan | -0.01 | -0.53 | -0.40 | -0.30 | -0.69 | -0.52 | -1.16 | 0.59 | 1.65 | -0.85 | 0.30 | -0.35 | -0.03 | 0.33 | -0.37 |
| Sikkim | -0.31 | -0.08 | 0.04 | -0.60 | -0.06 | 0.02 | 1.70 | -1.25 | -1.18 | 0.50 | 0.98 | 1.15 | 0.51 | 0.41 | -0.02 |
| Tamil Nadu | 0.28 | -0.53 | -0.06 | 0.02 | -0.63 | -0.12 | -0.56 | 0.39 | -1.82 | -0.68 | 0.49 | 0.47 | 0.58 | 0.28 | 1.54 |
| Telangana | 0.21 | -0.24 | -0.23 | 0.23 | -0.35 | -0.17 | -0.37 | -0.74 | 0.15 | -0.51 | 0.67 | 0.13 | 0.38 | 0.65 | 0.13 |
| Tripura | 0.24 | -0.48 | -0.36 | -0.53 | -0.54 | -0.26 | 0.22 | 0.24 | -0.55 | 0.46 | 0.66 | 0.54 | 0.23 | 0.12 | 0.62 |
| Uttar Pradesh | 0.25 | -0.61 | -0.33 | -0.36 | -0.45 | -0.39 | -0.25 | 0.17 | 0.04 | 0.48 | 0.50 | 0.48 | 0.05 | 0.40 | 0.21 |
| Uttarakhand | 0.00 | -0.01 | -0.36 | -0.38 | -0.12 | -0.55 | 0.98 | -0.32 | -1.48 | 0.43 | 0.15 | 1.40 | -0.55 | 0.29 | 1.00 |
| West Bengal | -0.35 | -0.47 | -0.17 | -0.33 | -0.47 | -0.53 | -0.19 | 0.37 | 1.37 | -0.49 | 0.41 | -0.57 | 0.18 | 0.16 | -0.10 |
| NCT Delhi (UT) | -0.01 | 0.01 | -0.26 | -0.12 | -0.13 | -0.46 | 0.33 | -0.60 | -0.92 | 0.42 | 0.51 | 0.75 | -0.01 | 0.21 | 0.89 |
| Jammu&Kashmir (UT) | -0.04 | -0.27 | -0.13 | -0.09 | -0.40 | -0.52 | 7.54 | -0.33 | 2.25 | 3.83 | 0.61 | -0.63 | 0.06 | 0.39 | -0.97 |
| Ladakh (UT) |  | -0.31 | -0.10 |  | -0.37 | -0.59 |  | 1.83 | -0.95 |  | -0.69 | 1.54 |  | -0.46 | 0.11 |
| Andaman&Nicobar (UT) |  |  | -0.12 |  |  | 0.03 |  |  | -0.67 |  |  | -0.20 |  |  | 0.97 |
| Chandigarh (UT) |  |  | -0.16 |  |  | 0.11 |  |  | -0.13 |  |  | -0.11 |  |  | 0.29 |
| Dadra and Nagar Haveli and Diu (UT) |  |  | 0.74 |  |  | 0.40 |  |  | -1.19 |  |  | -0.19 |  |  | 0.25 |
| Lakshadweep (UT) |  |  | -0.57 |  |  | -0.64 |  |  | 2.78 |  |  | 0.01 |  |  | -1.58 |
| Puducherry (UT) |  |  | -0.11 |  |  | -0.01 |  |  | -2.00 |  |  | 0.14 |  |  | 1.98 |

|  | **Severely/Moderately Thin** | | | **Mildly Thin** | | | **Normal** | | | **Overweight** | | | **Obese** | | |
| --- | --- | --- | --- | --- | --- | --- | --- | --- | --- | --- | --- | --- | --- | --- | --- |
|  | 1999-  2006 | 2006-2016 | 2016-  2021 | 1999-  2006 | 2006-  2016 | 2016-2021 | 1999-2006 | 2006-2016 | 2016-2021 | 1999-2006 | 2006-2016 | 2016-2021 | 1999-2006 | 2006-2016 | 2016-2021 |
| **Rural-India** | -0.17 | -0.79 | -0.52 | -0.17 | -0.80 | -0.66 | 0.44 | 0.70 | 0.13 | 0.16 | 0.68 | 0.72 | 0.08 | 0.21 | 0.34 |
| Andhra Pradesh | -0.20 | -1.09 | -0.28 | -0.23 | -0.97 | -0.59 | -4.86 | 0.16 | -0.16 | 0.62 | 1.33 | 0.50 | 0.09 | 0.56 | 0.53 |
| Arunachal Pradesh | 0.11 | -0.22 | -0.15 | 0.40 | -0.48 | -0.45 | 1.09 | -0.18 | -0.98 | 0.39 | 0.73 | 1.30 | 0.04 | 0.16 | 0.28 |
| Assam | 0.90 | -0.67 | -0.75 | 0.59 | -0.69 | -1.11 | -0.09 | 0.70 | 1.25 | 0.06 | 0.55 | 0.46 | -0.02 | 0.11 | 0.15 |
| Bihar | 0.54 | -0.99 | -0.39 | 0.31 | -0.84 | -0.86 | 0.70 | 0.99 | 0.09 | 0.05 | 0.66 | 0.88 | -0.02 | 0.18 | 0.27 |
| Chhattisgarh | -0.11 | -1.04 | -0.32 | -0.76 | -0.91 | -0.62 | -0.09 | 1.28 | 0.18 | -0.15 | 0.57 | 0.51 | -0.02 | 0.10 | 0.25 |
| Goa | 0.22 | -0.80 | -0.57 | -0.33 | -0.40 | -0.97 | 1.28 | -0.29 | 0.57 | 0.38 | 1.11 | 0.54 | 0.05 | 0.38 | 0.42 |
| Gujarat | -0.42 | -0.68 | -0.52 | -0.40 | -0.54 | -0.34 | -1.78 | 0.58 | 0.49 | 0.56 | 0.48 | 0.17 | 0.14 | 0.16 | 0.20 |
| Haryana | 0.35 | -0.92 | -0.23 | -0.15 | -0.95 | -0.25 | -0.80 | 1.20 | -2.32 | 0.32 | 0.52 | 1.72 | 0.20 | 0.15 | 1.08 |
| Himachal Pradesh | -0.44 | -0.67 | 0.02 | -0.19 | -0.72 | -0.44 | 0.83 | -0.36 | 0.06 | -0.05 | 1.19 | 0.28 | 0.01 | 0.57 | 0.08 |
| Jharkhand | 0.32 | -0.68 | -0.79 | 0.19 | -0.79 | -0.64 | 0.72 | 0.92 | 0.81 | 0.32 | 0.46 | 0.45 | -0.02 | 0.08 | 0.18 |
| Karnataka | -0.78 | -0.87 | -0.46 | -0.43 | -0.97 | -0.49 | -1.20 | 0.94 | -1.04 | 1.13 | 0.64 | 1.39 | 0.11 | 0.25 | 0.60 |
| Kerala | 0.06 | -0.51 | 0.03 | -0.71 | -0.36 | -0.12 | -0.53 | 0.27 | -0.93 | 0.10 | 0.44 | 0.50 | 0.24 | 0.16 | 0.52 |
| Madhya Pradesh | 0.57 | -0.85 | -0.68 | 0.31 | -0.79 | -0.84 | 0.77 | 0.91 | 0.60 | 0.48 | 0.57 | 0.68 | -0.02 | 0.16 | 0.24 |
| Maharashtra | -0.75 | -0.83 | -0.52 | -0.10 | -0.90 | -0.59 | -3.44 | 0.89 | 0.31 | 0.46 | 0.65 | 0.57 | 0.08 | 0.20 | 0.22 |
| Manipur | -0.45 | -0.12 | -0.04 | -0.18 | -0.66 | -0.23 | 0.53 | -0.57 | -1.69 | 0.08 | 1.04 | 1.43 | 0.05 | 0.31 | 0.52 |
| Meghalaya | -1.02 | -0.16 | -0.17 | -0.84 | -0.04 | -0.44 | 2.07 | -0.47 | 0.71 | 0.29 | 0.65 | -0.06 | 0.13 | 0.01 | -0.03 |
| Mizoram | -0.36 | -0.38 | -0.19 | -1.29 | -0.58 | -0.56 | 0.37 | 0.05 | -0.16 | -0.31 | 0.68 | 0.90 | -0.02 | 0.23 | 0.01 |
| Nagaland | -0.27 | -0.13 | -0.01 | -0.16 | -0.48 | -0.61 | 4.67 | -0.42 | 0.68 | 0.14 | 0.86 | 0.06 | -0.03 | 0.18 | -0.12 |
| Odisha | -0.27 | -0.88 | -0.64 | -0.66 | -0.84 | -0.61 | -0.50 | 0.68 | -0.05 | 0.50 | 0.85 | 1.00 | 0.06 | 0.19 | 0.30 |
| Punjab | -0.40 | -0.22 | 0.00 | -0.25 | -0.42 | 0.01 | -0.50 | 0.24 | -1.72 | 0.30 | 0.27 | 0.78 | 0.42 | 0.13 | 0.93 |
| Rajasthan | -0.14 | -0.42 | -0.85 | -0.30 | -0.70 | -0.97 | -2.51 | 0.42 | 1.91 | 0.23 | 0.56 | -0.04 | 0.05 | 0.15 | -0.04 |
| Sikkim | 0.05 | -0.17 | 0.13 | -0.16 | -0.38 | -0.15 | 2.60 | -0.49 | -1.49 | 0.62 | 0.88 | 1.19 | 0.07 | 0.15 | 0.32 |
| Tamil Nadu | -0.45 | -0.82 | -0.33 | -0.16 | -0.84 | -0.23 | 1.00 | 0.28 | -1.52 | 0.46 | 0.97 | 1.05 | 0.15 | 0.41 | 1.03 |
| Telangana | -0.55 | -0.64 | -0.64 | -0.70 | -0.61 | -0.74 | -0.90 | 0.35 | 0.24 | 0.29 | 0.74 | 0.73 | 0.32 | 0.16 | 0.41 |
| Tripura | 0.31 | -1.03 | -0.29 | -0.27 | -0.89 | -0.59 | 0.17 | 1.10 | -0.37 | 0.35 | 0.67 | 1.04 | -0.23 | 0.15 | 0.21 |
| Uttar Pradesh | -0.14 | -0.67 | -0.65 | -0.28 | -0.76 | -0.86 | -0.69 | 0.59 | 0.18 | 0.86 | 0.64 | 0.95 | 0.09 | 0.20 | 0.38 |
| Uttarakhand | 0.03 | -0.67 | -0.32 | -0.86 | -0.82 | -0.69 | 1.83 | 0.64 | -1.13 | 0.29 | 0.63 | 1.70 | 0.20 | 0.21 | 0.44 |
| West Bengal | -0.63 | -1.17 | -0.71 | -0.28 | -1.15 | -0.78 | -1.74 | 1.23 | 0.29 | -0.76 | 0.88 | 0.91 | 0.06 | 0.21 | 0.28 |
| NCT Delhi (UT) | 0.58 | -0.48 | -0.15 | -0.10 | -0.02 | -1.08 | 0.26 | -0.30 | -2.62 | 0.55 | 0.54 | 1.90 | 0.10 | 0.26 | 1.94 |
| Jammu&Kashmir (UT) | -0.28 | -0.62 | -0.49 | -0.36 | -0.87 | -1.05 | 8.67 | -0.01 | 0.82 | 1.59 | 1.00 | 1.23 | 0.10 | 0.50 | -0.51 |
| Ladakh (UT) |  | -0.83 | -0.19 |  | -0.88 | -1.18 |  | 1.40 | -1.73 |  | 0.33 | 2.83 |  | -0.03 | 0.27 |
| Andaman&Nicobar (UT) |  |  | -0.86 |  |  | -0.57 |  |  | -0.47 |  |  | 0.73 |  |  | 1.16 |
| Chandigarh (UT) |  |  | -1.00 |  |  | -3.00 |  |  | -5.00 |  |  | 6.00 |  |  | 3.00 |
| Dadra and Nagar Haveli and Diu (UT) |  |  | -0.44 |  |  | -0.49 |  |  | -0.74 |  |  | 1.29 |  |  | 0.39 |
| Lakshadweep (UT) |  |  | -0.67 |  |  | -0.59 |  |  | 2.02 |  |  | 0.95 |  |  | -1.71 |
| Puducherry (UT) |  |  | 0.10 |  |  | -0.23 |  |  | -1.96 |  |  | 0.84 |  |  | 1.24 |

**B. Men**

| <-1.00 | -1.00 to-0.76 | -0.75 to -0.51 | -0.50 to -0.26 | -0.25 to -0.01 | 0.00 to 0.25 | 0.26 to 0.50 | 0.51 to 0.75 | 0.76 to 1.00 | >1.00 |
| --- | --- | --- | --- | --- | --- | --- | --- | --- | --- |

|  | **Severely/Moderately Thin** | | **Mildly Thin** | | **Normal** | | **Overweight** | | **Obese** | |
| --- | --- | --- | --- | --- | --- | --- | --- | --- | --- | --- |
|  | 2006-2016 | 2016-2021 | 2006-2016 | 2016-2021 | 2006-2016 | 2016-2021 | 2006-2016 | 2016-2021 | 2006-2016 | 2016-2021 |
| All-India | -0.53 | -0.24 | -0.84 | -0.56 | 0.30 | -0.10 | 0.88 | 0.69 | 0.20 | 0.21 |
| Andhra Pradesh | -0.49 | -0.06 | -1.02 | 0.09 | -0.55 | 0.12 | 1.40 | 0.25 | 0.66 | -0.40 |
| Arunachal Pradesh | -0.17 | -0.27 | -0.53 | -0.49 | -0.75 | -0.95 | 1.21 | 1.43 | 0.25 | 0.28 |
| Assam | -0.58 | -0.32 | -0.84 | -1.24 | 0.57 | 0.65 | 0.73 | 0.78 | 0.12 | 0.13 |
| Bihar | -0.47 | -0.07 | -0.61 | -0.78 | 0.17 | 0.30 | 0.81 | 0.47 | 0.09 | 0.09 |
| Chhattisgarh | -0.35 | -0.34 | -0.99 | -0.78 | 0.82 | -0.07 | 0.46 | 0.96 | 0.07 | 0.24 |
| Goa | -0.68 | 0.08 | -0.52 | -0.37 | -0.70 | 0.66 | 1.21 | 0.67 | 0.70 | -1.03 |
| Gujarat | -0.57 | -0.19 | -0.67 | -0.43 | 0.36 | 0.80 | 0.67 | -0.24 | 0.21 | 0.06 |
| Haryana | -0.83 | 0.07 | -0.88 | 0.20 | 0.69 | -2.46 | 0.92 | 1.37 | 0.09 | 0.81 |
| Himachal Pradesh | -0.44 | -0.40 | -0.53 | -0.51 | -0.27 | -1.21 | 1.01 | 1.74 | 0.23 | 0.38 |
| Jharkhand | -0.48 | -0.52 | -0.94 | -0.83 | 0.68 | 0.41 | 0.66 | 0.85 | 0.09 | 0.08 |
| Karnataka | -0.66 | -0.18 | -0.97 | -0.34 | 0.33 | -1.12 | 1.06 | 1.20 | 0.24 | 0.43 |
| Kerala | -0.51 | 0.06 | -0.55 | -0.05 | 0.04 | -2.10 | 0.91 | 1.34 | 0.11 | 0.74 |
| Madhya Pradesh | -0.59 | -0.46 | -0.79 | -1.02 | 0.62 | 0.42 | 0.60 | 0.89 | 0.16 | 0.17 |
| Maharashtra | -0.61 | -0.07 | -0.77 | -0.40 | 0.10 | 0.37 | 1.00 | 0.06 | 0.29 | 0.03 |
| Manipur | -0.12 | -0.08 | -0.35 | -0.63 | -0.80 | -1.51 | 1.07 | 2.03 | 0.20 | 0.18 |
| Meghalaya | 0.05 | -0.15 | -0.15 | -0.44 | -0.42 | -0.33 | 0.37 | 0.88 | 0.16 | 0.04 |
| Mizoram | 0.04 | -0.16 | -0.15 | -0.35 | -1.03 | -2.07 | 0.69 | 2.29 | 0.45 | 0.29 |
| Nagaland | -0.15 | 0.02 | -0.19 | -0.62 | -0.62 | -1.92 | 0.87 | 2.10 | 0.10 | 0.42 |
| Odisha | -0.45 | -0.23 | -1.07 | -0.60 | 0.32 | -0.17 | 1.09 | 0.68 | 0.11 | 0.33 |
| Punjab | -0.29 | 0.06 | -0.41 | -0.06 | 0.10 | -1.11 | 0.45 | 0.41 | 0.14 | 0.69 |
| Rajasthan | -0.64 | -0.73 | -0.99 | -0.95 | 0.86 | 1.10 | 0.62 | 0.84 | 0.14 | -0.26 |
| Sikkim | -0.16 | 0.04 | -0.43 | -0.17 | -2.01 | 0.54 | 2.09 | -0.19 | 0.51 | -0.22 |
| Tamil Nadu | -0.57 | -0.03 | -0.77 | 0.00 | -0.25 | -1.79 | 1.35 | 0.88 | 0.25 | 0.94 |
| Telangana | -0.38 | -0.10 | -0.68 | -0.85 | -0.03 | -0.95 | 0.77 | 1.39 | 0.32 | 0.51 |
| Tripura | -1.16 | -0.15 | -1.07 | -0.67 | 1.03 | -0.57 | 0.99 | 1.17 | 0.21 | 0.22 |
| Uttar Pradesh | -0.43 | -0.70 | -0.84 | -0.93 | 0.65 | 0.23 | 0.56 | 1.07 | 0.06 | 0.33 |
| Uttarakhand | -0.55 | -0.05 | -0.67 | 0.02 | 0.21 | -2.47 | 0.86 | 1.88 | 0.16 | 0.62 |
| West Bengal | -0.55 | -0.47 | -1.01 | -0.54 | 0.61 | 0.47 | 0.80 | 0.51 | 0.15 | 0.03 |
| NCT Delhi (UT) | 0.11 | -0.40 | 0.04 | -1.26 | -1.26 | -0.56 | 0.90 | 1.66 | 0.20 | 0.56 |
| Jammu&Kashmir (UT) | -0.42 | -0.16 | -0.83 | -0.98 | -0.32 | -1.16 | 1.27 | 2.18 | 0.30 | 0.12 |
| Ladakh (UT) | -0.51 | -0.09 | -0.92 | -0.94 | 0.28 | -3.27 | 1.03 | 4.03 | 0.12 | 0.27 |
| Andaman&Nicobar (UT) |  | -0.20 |  | -0.35 |  | -0.20 |  | 0.77 |  | -0.02 |
| Chandigarh (UT) |  | -0.01 |  | -1.67 |  | 0.95 |  | -0.18 |  | 0.91 |
| Dadra and Nagar Haveli and Diu (UT) |  | 0.39 |  | -0.15 |  | 1.36 |  | -1.01 |  | -0.59 |
| Lakshadweep (UT) |  | 0.00 |  | -0.75 |  | -2.12 |  | 2.79 |  | 0.09 |
| Puducherry (UT) |  | -0.25 |  | 0.11 |  | -1.49 |  | 0.62 |  | 1.00 |

|  | **Severely/Moderately Thin** | | **Mildly Thin** | | **Normal** | | **Overweight** | | **Obese** | |
| --- | --- | --- | --- | --- | --- | --- | --- | --- | --- | --- |
|  | 2006-2016 | 2016-2021 | 2006-2016 | 2016-2021 | 2006-2016 | 2016-2021 | 2006-2016 | 2016-2021 | 2006-2016 | 2016-2021 |
| Urban-India | -0.41 | -0.17 | -0.57 | -0.27 | -0.17 | -0.17 | 0.90 | 0.37 | 0.24 | 0.24 |
| Andhra Pradesh | -0.01 | -0.03 | -0.56 | 0.55 | -1.78 | 0.64 | 1.75 | -0.61 | 0.81 | -0.55 |
| Arunachal Pradesh | -0.93 | 0.07 | -0.51 | -0.64 | -1.23 | -0.64 | 1.82 | 0.64 | 0.19 | 0.57 |
| Assam | -0.50 | -0.08 | -0.69 | -1.04 | 0.01 | 0.39 | 0.89 | 0.51 | 0.35 | 0.23 |
| Bihar | -0.41 | -0.25 | -0.54 | -0.75 | -0.41 | 1.57 | 1.42 | -0.22 | 0.14 | -0.34 |
| Chhattisgarh | -0.19 | -0.75 | -0.28 | -0.95 | 0.08 | 0.66 | 0.33 | 0.84 | 0.08 | 0.20 |
| Goa | -0.76 | 0.22 | -0.42 | -0.57 | -0.91 | 1.40 | 1.18 | 0.19 | 0.79 | -1.24 |
| Gujarat | -0.14 | -0.09 | -0.58 | -0.21 | 0.05 | 0.72 | 0.61 | -0.47 | 0.29 | 0.06 |
| Haryana | -0.21 | 0.15 | -0.59 | 0.01 | 0.99 | -3.00 | 0.20 | 1.90 | -0.09 | 0.94 |
| Himachal Pradesh | -0.76 | -0.64 | 0.06 | -1.39 | -1.21 | 0.08 | 0.89 | 0.94 | 0.29 | 1.01 |
| Jharkhand | -0.36 | -0.35 | -0.23 | -0.59 | -0.23 | 0.86 | 1.03 | 0.12 | 0.14 | -0.05 |
| Karnataka | -0.18 | -0.17 | -0.61 | -0.44 | -0.18 | -1.26 | 0.95 | 1.25 | 0.24 | 0.62 |
| Kerala | -0.88 | -0.03 | -0.60 | 0.06 | -0.01 | -2.46 | 0.86 | 1.88 | 0.15 | 0.55 |
| Madhya Pradesh | -0.20 | -0.47 | -0.53 | -0.59 | 0.05 | -0.64 | 0.54 | 1.40 | 0.33 | 0.30 |
| Maharashtra | -0.05 | 0.16 | -0.75 | 0.21 | -0.28 | 0.35 | 1.18 | -0.67 | 0.37 | -0.05 |
| Manipur | 0.18 | -0.05 | -0.03 | -1.01 | -0.76 | -1.53 | 0.68 | 2.48 | 0.21 | 0.11 |
| Meghalaya | 0.22 | -0.24 | -0.37 | -0.64 | -1.45 | -2.87 | 1.44 | 3.10 | 0.18 | 0.64 |
| Mizoram | -0.13 | -0.34 | -0.24 | -0.42 | -1.48 | -1.33 | 0.90 | 2.01 | 0.69 | 0.08 |
| Nagaland | -0.63 | 0.17 | -0.18 | -0.71 | -0.42 | -2.37 | 0.80 | 2.27 | 0.02 | 0.64 |
| Odisha | -0.23 | -0.01 | -0.61 | -0.36 | -0.77 | 0.27 | 1.88 | -0.63 | 0.00 | 0.73 |
| Punjab | -0.83 | 0.09 | -0.40 | -0.24 | 0.17 | -0.93 | 0.61 | 0.02 | -0.07 | 1.06 |
| Rajasthan | 0.15 | -0.61 | -0.60 | -0.59 | 0.60 | 1.05 | 0.36 | 0.75 | 0.20 | -0.60 |
| Sikkim | -0.68 | -0.08 | -0.36 | -0.08 | -2.07 | 1.49 | 1.76 | -1.47 | 0.88 | 0.13 |
| Tamil Nadu | -0.74 | 0.01 | -0.61 | 0.07 | -0.29 | -2.86 | 1.20 | 1.13 | 0.20 | 1.64 |
| Telangana | -0.03 | -0.14 | -0.21 | -0.75 | -0.55 | -1.74 | 0.62 | 1.73 | 0.38 | 0.90 |
| Tripura | -0.86 | 0.27 | -1.29 | -0.04 | 1.52 | -2.05 | 0.44 | 1.34 | 0.19 | 0.47 |
| Uttar Pradesh | -0.34 | -0.48 | -0.72 | -0.45 | 0.47 | 0.00 | 0.54 | 0.53 | 0.05 | 0.40 |
| Uttarakhand | -0.34 | -0.37 | -0.20 | 0.80 | -0.46 | -3.29 | 0.77 | 2.18 | 0.24 | 0.68 |
| West Bengal | -0.19 | -0.88 | -0.25 | -0.77 | -0.41 | 1.91 | 0.67 | -0.12 | 0.18 | -0.14 |
| NCT Delhi (UT) | 0.13 | -0.40 | 0.04 | -1.26 | -1.15 | -0.60 | 0.80 | 1.72 | 0.18 | 0.54 |
| Jammu&Kashmir (UT) | -0.38 | -0.02 | -0.56 | -0.77 | -0.96 | -1.58 | 1.46 | 2.05 | 0.44 | 0.33 |
| Ladakh (UT) | -0.39 | -0.11 | -0.03 | -1.63 | -0.46 | -3.02 | 0.76 | 4.16 | 0.13 | 0.60 |
| Andaman&Nicobar (UT) |  | -0.27 |  | 0.13 |  | 1.26 |  | 0.72 |  | -1.83 |
| Chandigarh (UT) |  | -0.08 |  | -0.85 |  | 0.71 |  | -0.58 |  | 0.80 |
| Dadra and Nagar Haveli and Diu (UT) |  | 0.84 |  | 0.09 |  | 0.53 |  | -1.06 |  | -0.40 |
| Lakshadweep (UT) |  | -0.60 |  | -0.78 |  | -1.71 |  | 2.88 |  | 0.22 |
| Puducherry (UT) |  | -0.36 |  | -0.14 |  | -0.77 |  | 0.63 |  | 0.65 |

|  | **Severely/Moderately Thin** | | **Mildly Thin** | | **Normal** | | **Overweight** | | **Obese** | |
| --- | --- | --- | --- | --- | --- | --- | --- | --- | --- | --- |
|  | 2006-2016 | 2016-2021 | 2006-2016 | 2016-2021 | 2006-2016 | 2016-2021 | 2006-2016 | 2016-2021 | 2006-2016 | 2016-2021 |
| Rural-India | -0.59 | -0.30 | -0.98 | -0.75 | 0.56 | -0.12 | 0.85 | 0.95 | 0.16 | 0.22 |
| Andhra Pradesh | -0.63 | -0.07 | -1.29 | -0.11 | 0.01 | -0.16 | 1.30 | 0.66 | 0.60 | -0.32 |
| Arunachal Pradesh | -0.14 | -0.36 | -0.54 | -0.44 | -0.60 | -1.19 | 1.01 | 1.78 | 0.27 | 0.22 |
| Assam | -0.58 | -0.37 | -0.90 | -1.29 | 0.68 | 0.67 | 0.74 | 0.87 | 0.07 | 0.12 |
| Bihar | -0.43 | -0.01 | -0.66 | -0.75 | 0.30 | 0.01 | 0.70 | 0.57 | 0.09 | 0.19 |
| Chhattisgarh | -0.40 | -0.22 | -1.22 | -0.75 | 1.07 | -0.34 | 0.49 | 1.05 | 0.06 | 0.26 |
| Goa | -0.72 | -0.16 | -0.62 | 0.00 | -0.35 | -0.63 | 1.15 | 1.48 | 0.54 | -0.68 |
| Gujarat | -0.70 | -0.30 | -0.71 | -0.63 | 0.60 | 0.85 | 0.67 | -0.01 | 0.14 | 0.08 |
| Haryana | -0.96 | 0.00 | -1.00 | 0.26 | 0.54 | -2.18 | 1.26 | 1.13 | 0.16 | 0.78 |
| Himachal Pradesh | -0.51 | -0.36 | -0.62 | -0.36 | -0.11 | -1.44 | 1.02 | 1.88 | 0.22 | 0.28 |
| Jharkhand | -0.39 | -0.60 | -1.27 | -0.93 | 1.06 | 0.23 | 0.53 | 1.16 | 0.07 | 0.14 |
| Karnataka | -0.84 | -0.19 | -1.21 | -0.33 | 0.73 | -1.18 | 1.10 | 1.34 | 0.23 | 0.36 |
| Kerala | -0.56 | 0.13 | -0.50 | -0.14 | 0.13 | -1.80 | 0.87 | 0.91 | 0.07 | 0.91 |
| Madhya Pradesh | -0.66 | -0.49 | -0.87 | -1.31 | 0.86 | 0.73 | 0.59 | 0.88 | 0.07 | 0.19 |
| Maharashtra | -0.73 | -0.29 | -0.84 | -0.95 | 0.43 | 0.23 | 0.91 | 0.85 | 0.22 | 0.16 |
| Manipur | -0.14 | -0.12 | -0.52 | -0.37 | -0.77 | -1.39 | 1.25 | 1.67 | 0.19 | 0.22 |
| Meghalaya | 0.00 | -0.13 | -0.08 | -0.40 | -0.14 | 0.12 | 0.07 | 0.49 | 0.15 | -0.09 |
| Mizoram | -0.09 | 0.07 | -0.02 | -0.29 | -0.36 | -3.20 | 0.36 | 2.79 | 0.12 | 0.63 |
| Nagaland | -0.13 | -0.07 | -0.19 | -0.56 | -0.67 | -1.59 | 0.86 | 1.93 | 0.13 | 0.29 |
| Odisha | -0.44 | -0.29 | -1.17 | -0.66 | 0.58 | -0.29 | 0.88 | 1.01 | 0.14 | 0.24 |
| Punjab | -0.27 | 0.05 | -0.42 | 0.07 | 0.01 | -1.24 | 0.38 | 0.69 | 0.31 | 0.44 |
| Rajasthan | -0.68 | -0.80 | -1.19 | -1.12 | 0.97 | 1.10 | 0.77 | 0.93 | 0.13 | -0.11 |
| Sikkim | -0.14 | 0.11 | -0.40 | -0.25 | -1.79 | -0.18 | 2.10 | 0.64 | 0.23 | -0.33 |
| Tamil Nadu | -0.65 | -0.08 | -0.92 | -0.09 | -0.20 | -1.02 | 1.48 | 0.79 | 0.29 | 0.41 |
| Telangana | -0.43 | -0.14 | -0.89 | -1.01 | 0.33 | -0.80 | 0.74 | 1.49 | 0.25 | 0.46 |
| Tripura | -1.19 | -0.34 | -0.98 | -0.96 | 0.93 | 0.09 | 1.01 | 1.10 | 0.22 | 0.11 |
| Uttar Pradesh | -0.46 | -0.82 | -0.86 | -1.22 | 0.73 | 0.25 | 0.53 | 1.43 | 0.05 | 0.35 |
| Uttarakhand | -0.67 | 0.12 | -0.90 | -0.39 | 0.62 | -2.33 | 0.86 | 1.96 | 0.10 | 0.64 |
| West Bengal | -0.72 | -0.27 | -1.41 | -0.42 | 1.09 | -0.19 | 0.90 | 0.78 | 0.15 | 0.11 |
| NCT Delhi (UT) | -0.48 | 0.00 | -0.81 | 0.00 | -6.48 | 7.06 | 7.78 | -9.58 | 0.00 | 2.52 |
| Jammu&Kashmir (UT) | -0.43 | -0.23 | -0.95 | -1.12 | 0.01 | -1.22 | 1.14 | 2.46 | 0.23 | 0.11 |
| Ladakh (UT) | -0.57 | -0.08 | -1.37 | -0.51 | 0.74 | -3.96 | 1.10 | 4.27 | 0.10 | 0.28 |
| Andaman&Nicobar (UT) |  | -0.14 |  | -0.69 |  | -1.23 |  | 0.81 |  | 1.26 |
| Chandigarh (UT) |  | 0.00 |  | -10.00 |  | -7.50 |  | -2.50 |  | 0.00 |
| Dadra and Nagar Haveli and Diu (UT) |  | -0.26 |  | -0.71 |  | 1.47 |  | 0.04 |  | -0.54 |
| Lakshadweep (UT) |  | 1.88 |  | -0.66 |  | -3.49 |  | 2.87 |  | -0.60 |
| Puducherry (UT) |  | 0.02 |  | 0.74 |  | -3.03 |  | 0.38 |  | 1.88 |

**Figure S5: Relationship between the prevalence and Headcount Burden of Normal BMI, 2021**

A. Women

B. Men

**Note:** AN: Andaman & Nicobar, AP: Andhra Pradesh, AR: Arunachal Pradesh, AS: Assam, BR: Bihar, CH: Chandigarh, CG: Chhattisgarh, DH: Dadra and Nagar Haveli and Daman and Diu, GA: Goa, GJ: Gujarat, HR: Haryana, HP: Himachal Pradesh, JK: Jammu & Kashmir, JH: Jharkhand, KA: Karnataka, KL: Kerala, LK: Ladakh, LD: Lakshadweep, MP: Madhya Pradesh, MH: Maharashtra, MN: Manipur, ML: Meghalaya, MZ: Mizoram, DL: NCT Delhi, NL: Nagaland, OR: Odisha, PY: Puducherry, PB: Punjab, RJ: Rajasthan, SK: Sikkim, TN: Tamil Nadu, TL: Telangana, TR: Tripura, UP: Uttar Pradesh, UK: Uttarakhand, WB: West Bengal.
